# TET2 suppresses vascular calcification by forming an inhibitory complex with HDAC1/2 and SNIP1 independent of demethylation

**DOI:** 10.1172/JCI186673

**Published:** 2025-03-11

**Authors:** Dayu He, Jianshuai Ma, Ziting Zhou, Yanli Qi, Yaxin Lian, Feng Wang, Huiyong Yin, Huanji Zhang, Tingting Zhang, Hui Huang

**Affiliations:** 1 Department of Cardiology, Joint Laboratory of Guangdong–Hong Kong–Macao Universities for Nutritional Metabolism and Precise Prevention and Control of Major Chronic Diseases, Eighth Affiliated Hospital of Sun Yat-sen University, Shenzhen, China.; 2 Sixth Hospital of Wuhan, Affiliated Hospital of Jianghan University, Wuhan, China.; 3 Department of Biomedical Sciences, Tung Biomedical Science Center, Shenzhen Research Institute and Futian Research Institute, College of Biomedicine, City University of Hong Kong, Hong Kong, China.

**Keywords:** Cardiology, Vascular biology, Cardiovascular disease

## Abstract

Osteogenic transdifferentiation of vascular smooth muscle cells (VSMCs) has been recognized as the principal mechanism underlying vascular calcification (VC). Runt-related transcription factor 2 (RUNX2) in VSMCs plays a pivotal role because it constitutes an osteogenic transcription factor essential for bone formation. As a key DNA demethylation enzyme, ten-eleven translocation 2 (TET2) is crucial in maintaining the VSMC phenotype. However, whether TET2 involves in VC progression remains elusive. Here we identified a substantial downregulation of TET2 in calcified human and mouse arteries, as well as human primary VSMCs. In vitro gain- and loss-of-function experiments demonstrated that TET2 regulated VC. Subsequently, in vivo knockdown of TET2 significantly exacerbated VC in both vitamin D3– and adenine diet–induced chronic kidney disease (CKD) mouse models. Mechanistically, TET2 bound to and suppressed activity of the P2 promoter within the RUNX2 gene; however, an enzymatic loss-of-function mutation of TET2 did not change its binding and suppressive effects. Furthermore, TET2 formed a complex with histone deacetylases 1/2 (HDAC1/2) to deacetylate H3K27ac on the P2 promoter, thereby inhibiting its transcription. Moreover, SNIP1 was indispensable for TET2 to interact with HDAC1/2 to exert an inhibitory effect on VC, and knockdown of SNIP1 accelerated VC in mice. Collectively, our findings imply that TET2 might serve as a potential therapeutic target for VC.

## Abstract 2

<strong>TET2 is a key inhibitor of vascular calcification by forming inhibitory complexes with other regulators to suppress osteoblast genes expression.</strong>

## Graphical abstract

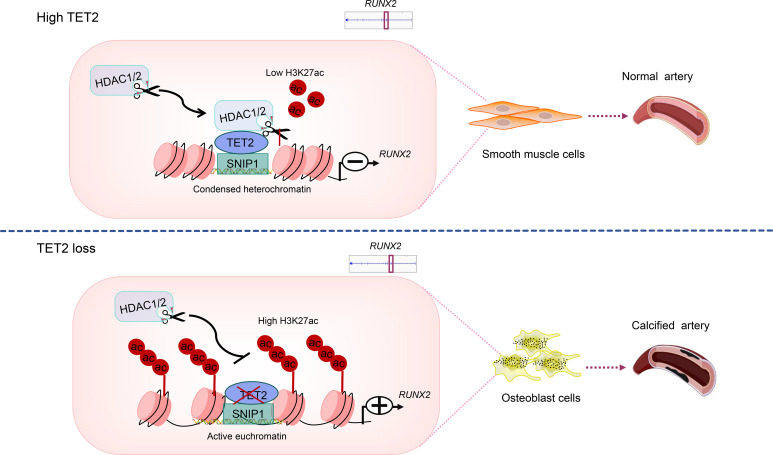

## Introduction

Vascular calcification (VC), which constitutes a severe complication of chronic kidney disease (CKD) (1) as well as diabetes mellitus (2), plays a significant role in contributing to high cardiovascular morbidity and mortality (3, 4). Among the various underlying pathogeneses that have been established in recent years, osteogenic transdifferentiation of vascular smooth muscle cells (VSMCs) bears the key responsibility (5–8). Unlike most mature cells, VSMCs are able to undergo plastic changes in response to environmental stimuli. VSMC osteogenic transdifferentiation is one such pattern, characterized by the downregulation of VSMC markers and the concurrent upregulation of osteogenic genes (9–11). The plasticity of VSMCs renders gene regulation a rather complex process. Several transcription factors have been identified, and among them, runt-related transcription factor 2 (RUNX2) has been demonstrated to be a necessary and sufficient regulator of VSMC osteogenic differentiation (12, 13). Previous studies have disclosed a causal role of RUNX2 in promoting osteogenic transdifferentiation of VSMCs (14–16). Moreover, it has been observed that RUNX2 expression remains low in healthy vasculature but is remarkably increased in calcified arteries of animal models and in humans with CKD, atherosclerosis, and diabetes mellitus (17–20). Furthermore, specific deletion of RUNX2 in VSMCs within mouse models has indicated that loss of RUNX2 can inhibit VC (19, 21). It is worth noting that RUNX2 deletion did not lead to any alterations in the VSMC phenotype or the normal development of the vascular (19, 21). However, a synergetic mechanism that collaboratively governs the VSMC contractile phenotype and osteogenic transdifferentiation has yet to be uncovered.

The ten-eleven translocation (TET) family of proteins, including TET1, TET2, and TET3 in mammalian cells, oxidize 5-methylcytosine (5-mC) to generate 5-hydroxymethylcytosine (5-hmC) (22). Pathologically, TET2 exhibits a close association with a spectrum of cardiovascular diseases (23, 24). Specifically, patients harboring a TET2 mutation or experiencing a loss of TET2 functionality are predisposed to an elevated risk of developing various cardiovascular pathologies, such as atherosclerosis (25), pulmonary hypertension (23), aortic valve stenosis, and heart failure (26, 27). It is noteworthy that for the majority of these diseases, the incidence of comorbidity with VC is high, highlighting the potential interplay between TET2 disorders and the manifestation of VC. Furthermore, studies revealed that TET2 is a master epigenetic regulator of VSMC differentiation, and loss of TET2 leading to VSMC dedifferentiation (28). As has been demonstrated, VSMCs are able to undergo transdifferentiation into alternative cell phenotypes, such as a macrophage, synthetic, or osteogenic phenotype (10). However, the role of TET2 in VSMC osteogenic transdifferentiation and its specific mechanisms have remained unclear. In this study, we explored the role of TET2 in VC. We observed a marked downregulation of TET2 in groups with calcification in both clinical settings and mouse models. Further gain- and loss-of-function experiments revealed the protective role of TET2 in VSMC osteogenic transdifferentiation. Mechanistically, our investigations uncovered that TET2 plays a crucial and necessary role in the inhibition of *RUNX2* gene transcription. This is achieved by formation of an inhibitory complex in conjunction with other regulatory factors. The formation of such a complex constitutes a key regulatory mechanism that intervenes in the transcriptional process of the *RUNX2* gene, which is a well-documented driver of osteogenic transdifferentiation in VSMCs. Most importantly, we have illustrated the existence of an epigenetic regulator that functions in a synergistic manner to regulate both contractile and osteogenic genes within VSMCs. It is hoped that this discovery will enrich our understanding of the intricate gene-regulatory network governing VSMC phenotype and function. Above all, we demonstrated a key role for TET2 in VC, with results suggesting the potential for targeting the TET2-HDAC1/2-SNIP1 complex pathway to inhibit VSMC osteogenic differentiation.

## Results

### TET2 is negatively correlated with VC in both human and mouse specimens.

**Figure 1:**
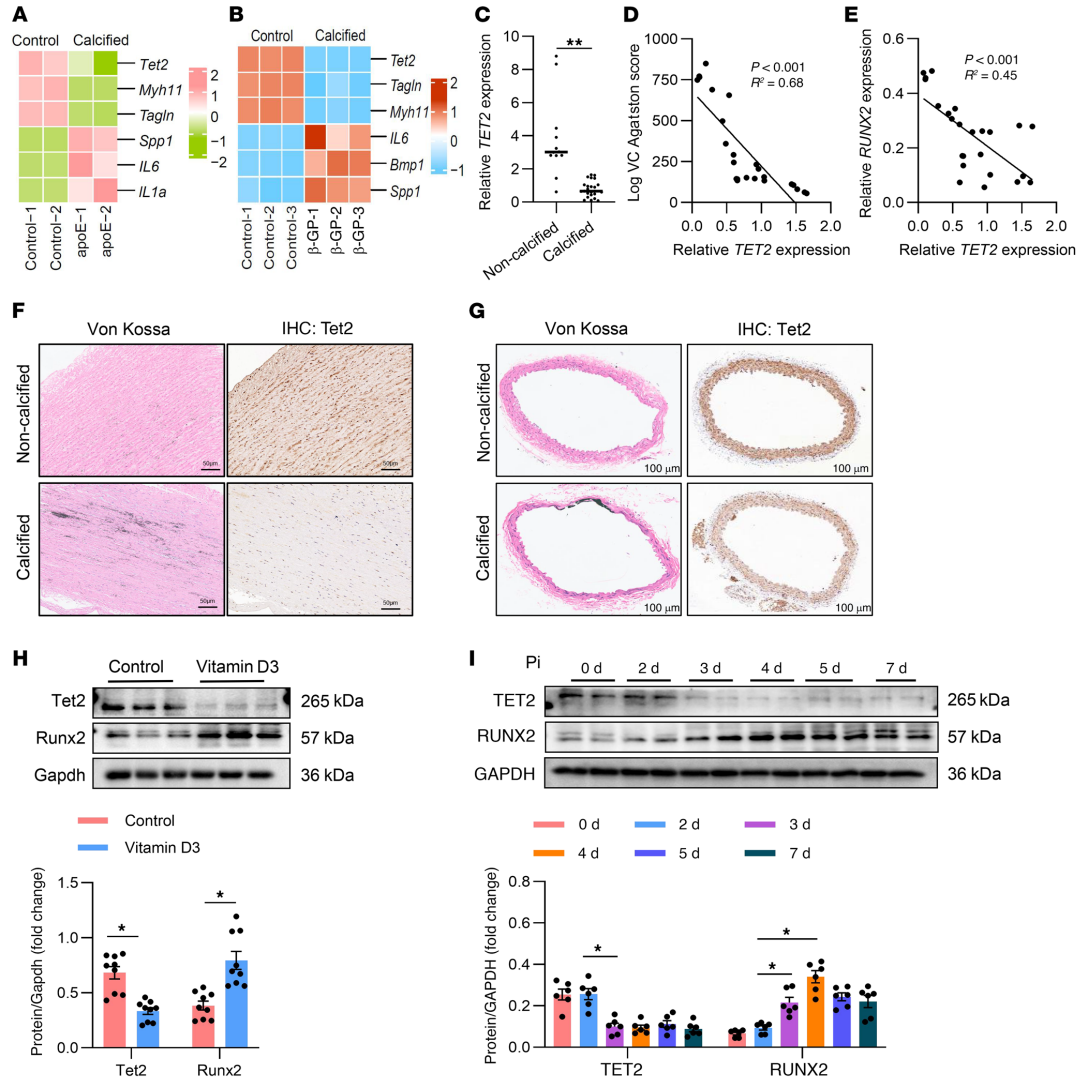
TET2 is negatively correlated with VC in both human and mouse specimens. (**A** and **B**) Heatmap showing *TET2*, SMC marker (*Myh11, Tagln*), and osteogenic marker (*Spp1, IL6, IL1a, Bmp1*) mRNA expression in control and *apoE^–/–^* calcified mouse aorta (**A**) and β-GP treatment–induced calcified mouse aorta (**B**). (**C**) Leukocyte *TET2* mRNA expression in patients with CKD with calcified (*n* = 21) or noncalcified arteries (*n* = 12). (**D**) Correlation between leukocyte *TET2* mRNA expression and calcific score (**D**), or *RUNX2* mRNA expression (**E**) in CKD patients with calcification (VC, *n* = 21). (**F**) Von Kossa staining and immunohistochemical images of TET2 expression in control and calcified arteries from patients with CKD. Scale bars: 50 μm. *n* = 6. (**G**) Von Kossa staining and immunohistochemical images of Tet2 expression in control and calcified mouse arteries. Scale bars: 100 μm. *n* = 3. (**H** and **I**) Western blot analysis and quantification of TET2 and RUNX2 expression in calcified mouse and control arteries (**H**) (*n* = 3) or in hVSMCs induced by Pi for the indicated time (**I**) (*n* = 3). All values are presented as mean ± SEM. *P < 0.05, **P < 0.01. Statistical significance was assessed using 2-tailed *t* tests (**C**), 1-way ANOVA followed by Dunnett’s test (**H** and **I**), and Pearson’s correlation coefficient analysis (**D** and **E**).

To investigate the role of TET2 in VC, we downloaded publicly available data of high-throughput sequencing from the Gene Expression Omnibus database (GEO GSE159832 and GSE254077). Heatmaps were utilized to show the expression of *Tet2*, osteogenic genes (*Spp1, IL6, IL1a, and Bmp1*), and VSMC phenotype genes (*Myh11, Tagln*) in apolipoprotein E–knockout (*ApoE^–/–^*) mouse aorta (atherosclerotic calcification) and β-glycerophosphate (β-GP) treatment–induced calcified mouse aorta. As depicted in the heatmaps, compared with normal controls, expression of TET2 was substantially decreased in the *ApoE^–/–^* mouse aorta with atherosclerotic calcification lesion (Figure 1A) and β-GP treatment–induced calcified mouse aorta (Figure 1B). This suggested a crucial role of TET2 in VC. Furthermore, human leucocyte *TET2* mRNA levels were evaluated in patients with CKD with VC (*n* = 24) and CKD without VC (*n* = 12). The basic characteristics of patients are shown in Supplemental Table 1 (supplemental material available online with this article; https://doi.org/10.1172/JCI186673DS1). As is shown in Figure 1C, compared with those in the noncalcified groups, *TET2* mRNA levels were significantly decreased in patients with VC (95% CI [8.258,4.024], *P* < 0.001). *TET2* mRNA levels were negatively related to calcific score (*r²* = 0.68, *P* < 0.001) (Figure 1D) and *RUNX2* mRNA levels (*r²* = 0.45, *P* < 0.001) (Figure 1E). We also detected *TET2* mRNA levels in healthy people (*n* = 21), finding that *TET2* significantly decreased both in CKD patients with and without VC (Supplemental Figure 1, A and B). Then we detected TET2 levels in calcified and noncalcified human arteries. The calcified arteries were collected from patients with CKD undergoing arterial venous fistula operation and diagnosed with aortic arch calcification (CKD, *n* = 6). Control arteries were obtained from patients who underwent amputation surgery due to upper limb trauma, without a diagnosis of CKD or diabetes mellitus (control, *n* = 6). Both immunofluorescence (IF) staining (Supplemental Figure 1C) and IHC staining (Figure 1F) revealed that TET2 expression was substantially decreased in calcified human arteries. Furthermore, we tested TET2 levels in calcified aorta of mice injected with vitamin D3. Results of both IHC (Figure 1G) and Western blot analysis (Figure 1H) confirmed a marked downregulation of TET2 in the calcified mouse aorta. Then we detected TET2 expression in human primary aorta VSMCs (hVSMCs) with high inorganic phosphate–induced (Pi-induced) calcification and discovered that TET2 significantly declined as Pi treatment time increased (Figure 1I).

### TET2 plays a role in human primary VSMC osteogenic transdifferentiation.

**Figure 2:**
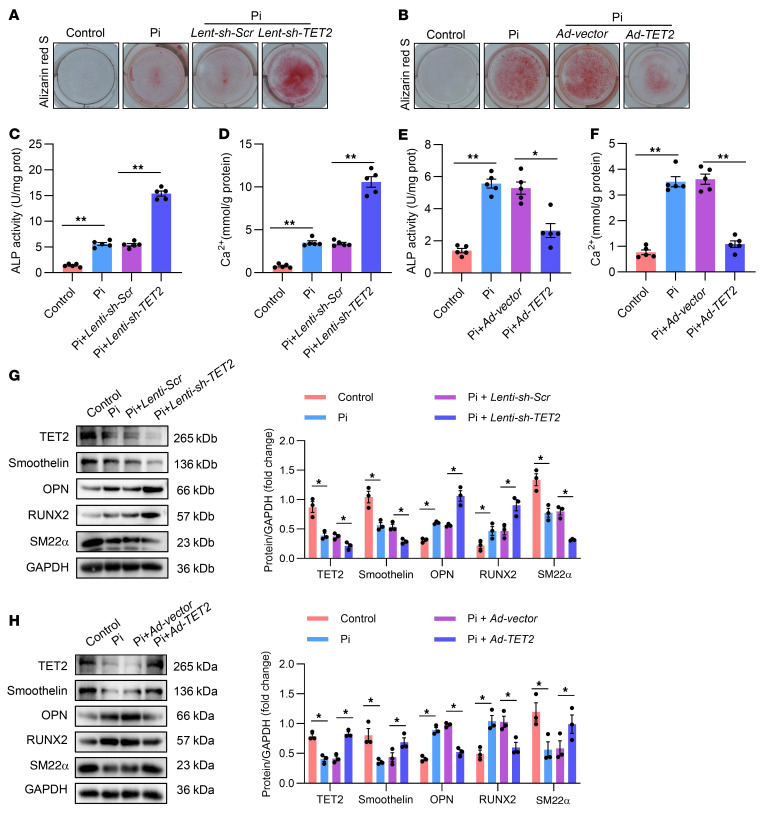
TET2 plays a role in human primary VSMC osteogenic transdifferentiation. (**A** and **B**) Alizarin red staining of hVSMCs transfected with *Lenti-sh-Scr* or with *Lenti-sh-TET2* (**A**) and transfected with *Ad-Vector* or *Ad-TET2* (**B**) (*n* = 3). (**C** and **D**) ALP activity assay (**C**) and quantification of calcium content (**D**) in hVSMCs transfected with *Lenti-sh-Scr* or *Lenti-sh-TET2* (*n* = 5). (**E** and **F**) ALP activity assay (**E**) and quantification of calcium content (**F**) in hVSMCs transfected with *Ad-Vector* or *Ad-TET2* (*n* = 5). (**G** and **H**) Western blot analysis and quantification of TET2, RUNX2, OPN, smoothelin, and SM22α expression in hVSMCs transfected with *Lenti-sh-Scr* or with *Lenti-sh-TET2* (**G**) and transfected with *Ad-Vector* or *Ad-TET2* (**H**) (*n* = 3). All values are presented as mean ± SEM. *P < 0.05, **P < 0.01. Statistical significance was assessed using 1-way ANOVA followed by Dunnett’s test (**C**–**H**).

To assess the causal role of TET2 in hVSMC osteogenic reprogramming, we constructed a lentivirus carrying shRNA specific to the human *TET2* gene (*shTET2*) (Supplemental Table 6). As shown by Western blot data, TET2 was substantially knocked down in hVSMCs (Supplemental Figure 3A). Results indicated that depletion of TET2 significantly exacerbated hVSMC calcification, which was ascertained through alizarin red S staining (Figure 2A), quantification of alkaline phosphatase (ALP) activity (Figure 2C), and calcium assay (Figure 2D). Further Western blot analysis disclosed substantial upregulation of expression of osteogenic differentiation genes, including OPN and RUNX2, while there was marked downregulation of VSMC phenotype genes, including smoothelin and SM22α (Figure 2G). Conversely, overexpression of TET2 via adenovirus markedly mitigated the Pi-induced hVSMC calcification. This was gauged by alizarin red S staining (Figure 2B), quantification of ALP activity (Figure 2E), and calcium assay (Figure 2F). The Western blot analysis showed a significant downregulation in expression of osteogenic differentiation genes, including OPN and RUNX2, while there was a marked upregulation of VSMC phenotype genes, including smoothelin and SM22α (Figure 2H). Taken together, these results suggest that TET2 serves to inhibit the osteogenic transdifferentiation of hVSMCs.

### VSMC specific loss of Tet2 in mice aggravated VC.

**Figure 3:**
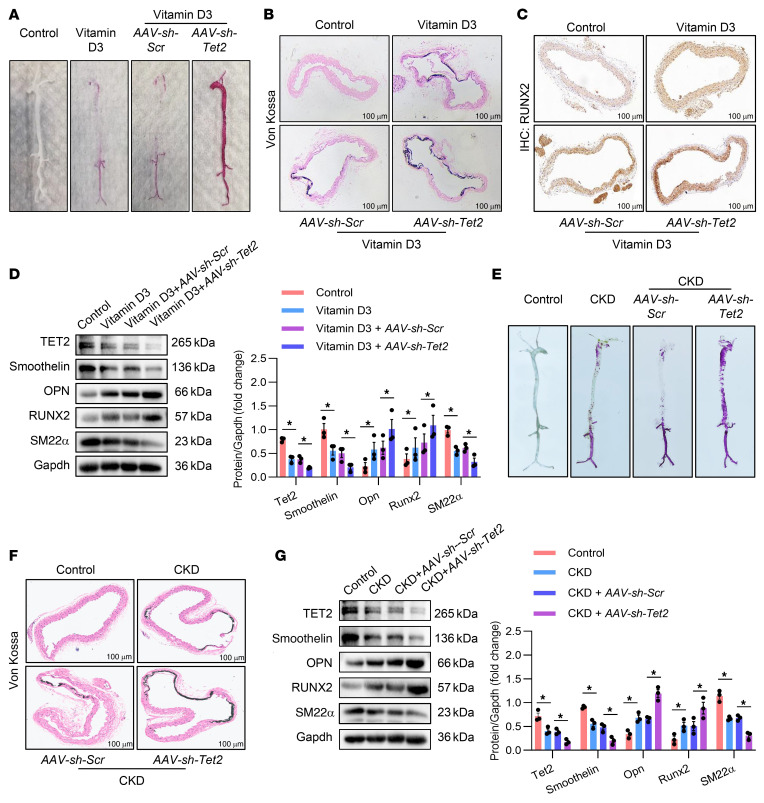
VSMC-specific loss of Tet2 in mice aggravated VC. (**A**) Representative alizarin red S staining images of whole aortas from control mice, mice injected with vitamin D3, and mice injected with vitamin D3 together with *AAV-sh-Scr* or *AAV-sh-Tet2*. *n* = 3. (**B**) Representative von Kossa staining of aortic sections from control mice, mice injected with vitamin D3, and mice injected with vitamin D3 together with *AAV-sh-Scr* or *AAV-sh-Tet2*. Scale bars: 100 μm. *n* = 3. (**C**) Representative immunohistochemical images of Runx2 expression in aortic sections from control mice, mice injected with vitamin D3, and mice injected with vitamin D3 together with *AAV-sh-Scr* or *AAV-sh-Tet2*. Scale bars: 100 μm. *n* = 3. (**D**) Western blot analysis and quantification of Tet2 and osteogenic phenotypic marker (Runx2 and Opn) and contractile phenotype marker (smoothelin and SM22α) expression in aortas from control mice, mice injected with vitamin D3, and mice injected with vitamin D3 together with *AAV-sh-Scr* or *AAV-sh-Tet2*. *n* = 3. (**E**) Representative alizarin red S staining images of whole aortas from control mice, CKD model mice, and CKD model mice injected with *AAV-sh-Scr* or *AAV-sh-Tet2*. *n* = 3. (**F**) Representative von Kossa staining of aortic sections from control mice, CKD model mice, and CKD model mice injected with AAV-sh-Scr or AAV-sh-Tet2. Scale bars: 100μm. *n* = 3. (**G**) Western blot analysis and quantification of Tet2 and osteogenic phenotypic marker (Runx2 and Opn) and contractile phenotype marker (smoothelin and SM22α) expression in aortas from control mice, CKD model mice, and CKD model mice injected with AAV-sh-Scr or AAV-sh-Tet2. *n* = 3. All values are presented as mean ± SEM. *P < 0.05, **P < 0.01. Statistical significance was assessed using 1-way ANOVA followed by Dunnett’s test (**D** and **G**).

In order to investigate the potential role of Tet2 in vivo, we employed adeno-associated virus 9 (AAV9) with the transgelin (TAGLN) promoter to achieve VSMC-specific knockdown of Tet2 in mice. The sequences for AAV with scrambled shRNA (*AAV-sh-Scr*) or *Tet2* shRNA (*AAV-sh-Tet2*) is presented in Supplemental Table 7. Knockdown efficiency of Tet2 in the aorta was evaluated by Western blot analysis (Supplemental Figure 3B). We constructed both a vitamin D3 model and an adenine diet–induced model of CKD to investigate the role of Tet2 in in vivo calcification. We tested several key systemic parameters, including those related to liver function, such as alanine transaminase (ALT) and aspartate transaminase (AST); as well as those associated with renal function, such as serum urea nitrogen and creatinine. We also examined systemic metabolism parameters such as calcium levels and body weight. Our findings revealed that there were no significant difference in these characteristics between the *AAV-sh-Scr* *AAV-sh-Tet2* groups (Supplemental Tables 2 and 3). However, it was notable that the level of serum ALP was significantly elevated in the *AAV-sh-Tet2* groups (Supplemental Tables 2 and 3). As depicted in Figure 3, A and E, the loss of Tet2 markedly aggravated calcium deposition and mineralization in the aorta compared with the *sh-Scr* controls, as confirmed by alizarin red S staining. Consistent with these findings, von Kossa staining demonstrated substantially increased calcium deposition in the aortic sections of Tet2-knockdown mice (Figure 3, B and F). IHC staining indicated substantially higher Runx2 expression in the *AAV-sh-Tet2* than in the *AAV-sh-Scr* groups (Figure 3C). Moreover, Western blot and quantification analysis revealed that, in contrast to the *AAV-sh-Scr* groups, the *AAV-sh-Tet2* groups exhibited significantly higher expression of the osteogenic markers Runx2 and Opn, while levels of the VSMC contractile markers smoothelin and SM22α were lower (Figure 3, D and G). Collectively, our results of experiments using two distinct calcified models validated the effects of Tet2 on calcification.

### TET2 inhibits RUNX2 gene transcription not by DNA demethylation but by decreasing H3K27ac on the P2 promoter.

**Figure 4:**
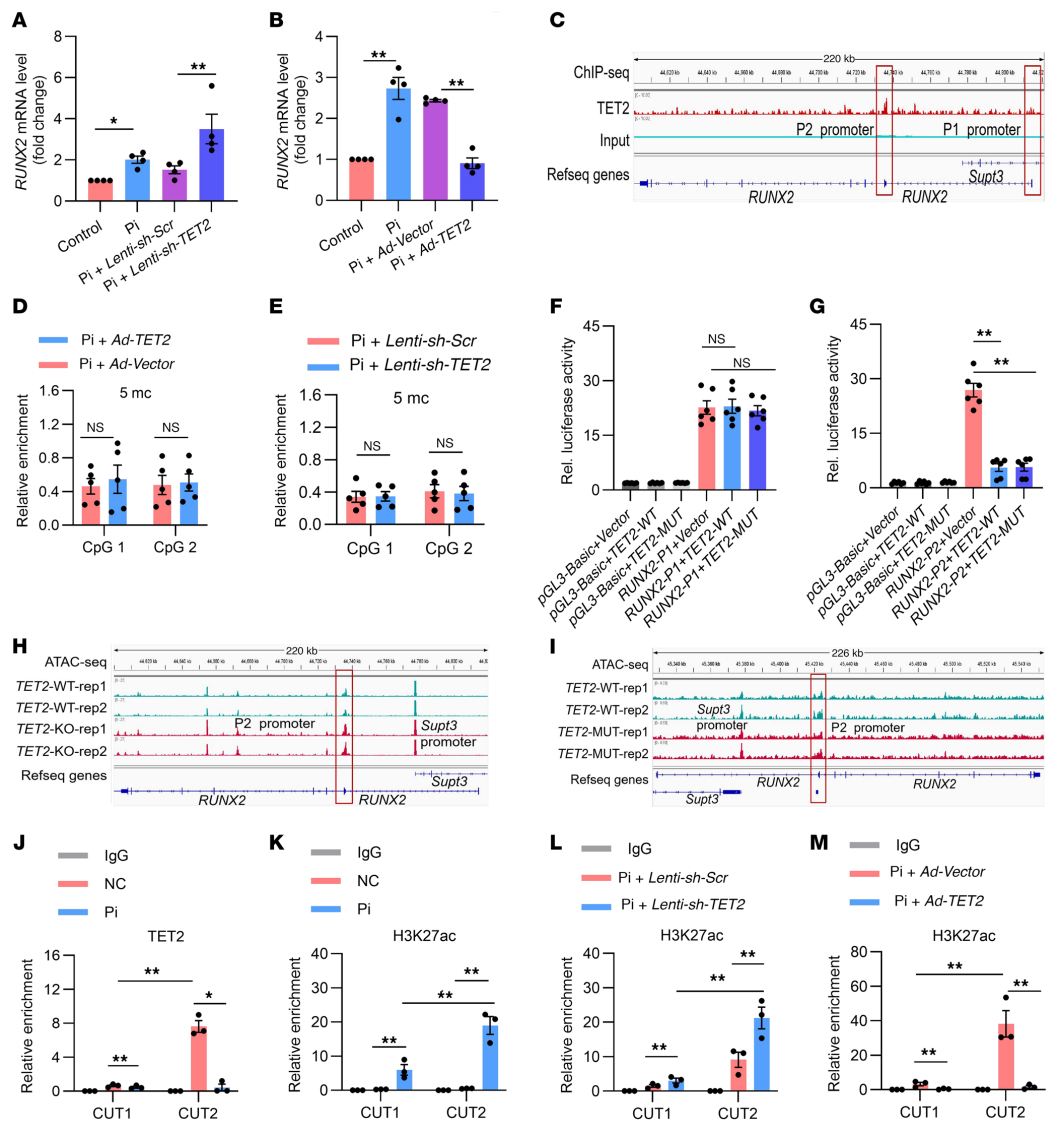
TET2 inhibits *RUNX2* gene transcription not by DNA demethylation but by decreasing H3K27ac on the P2 promoter. (**A** and **B**) Quantitative real-time PCR analysis of *RUNX2* expression in hVSMCs transfected with *Lenti-sh-Scr* or *Lenti-sh-TET2* (**A**), or *Ad-Vector* or *Ad-TET2* (**B**) (*n* = 4). (**C**) ChiP-Seq analysis for TET2 enrichment on the *RUNX2* gene. (**D** and **E**) DNA methylation quantified by MethylCap-qPCR in the *RUNX2* P2 promoter from hVSMCs with TET2 overexpression (**D**) or TET2 knockdown (**E**) (*n* = 5). (**F** and **G**) Luciferase activity analyzed after cotransfection with control *Renilla* luciferase plasmid and constructs of the *RUNX2* P1 promoter (**F**) or P2 promoter–driven luciferase reporters (**G**), and cotransfection with control, *TET2*-WT, or enzyme activity locus**–**mutated *TET2* (*TET2*-MUT) (*n* = 6). (**H**) ATAC-Seq analysis for *RUNX2* gene transposase-accessible chromatin in the *TET2*-WT and *TET2*-KO groups. (**I**) ATAC-Seq analysis for *RUNX2* gene transposase-accessible chromatin in the *TET2*-WT and *TET2*-MUT groups. (**J** and **K**) TET2 CUT&Tag-qPCR (**J**) and H3K27ac CUT&Tag-qPCR (**K**) at the *RUNX2* (CUT1) P1 and (CUT2) P2 promoter in either control or Pi-exposed hVSMCs (*n* = 3). (**L** and **M**) H3K27ac CUT&Tag-qPCR at the *RUNX2* (CUT1) P1 and (CUT2) P2 promoter in hVSMCs with either TET2 knockdown (**L**) or TET2 overexpression (**M**) (*n* = 3). All values are presented as mean ± SD. *P < 0.05, **P < 0.01. Statistical significance was assessed using 1-way ANOVA followed by Dunnett’s test (**A**, **B**, **D**–**G** and **J**–**M**).

To investigate the specific mechanisms underlying the role of TET2 in VC, and given the crucial role of RUNX2 in VC, we initially carried out quantitative PCR (qPCR) experiments to analyze mRNA levels of *RUNX2*. As is depicted in Figure 4, A and B, the mRNA level of *RUNX2* was significantly upregulated in the TET2-knockdown groups (Figure 4A), while it was substantially inhibited in the TET2-overexpressed groups (Figure 4B). To examine whether TET2 regulates *RUNX2* gene transcription, we analyzed the TET2-enriched chromatin based on the data from ChIP sequencing (ChIP-Seq). It is known that *RUNX2* gene transcription is governed by two promoters, namely the distal P1 promoter and the proximal P2 promoter, which encode two major isoforms via exons 1–8 (type II) or exons 2–8 (type I) (Supplemental Figure 2) (29, 30). The results of ChiP-Seq demonstrated that TET2 peaks were distributed across the *RUNX2* genome, with a substantially higher enrichment on the P2 promoter compared with the P1 promoter (Figure 4C) (31). Considering that TET2 is recognized to contribute to DNA demethylation (22, 28), we tested 5-mC levels in the *RUNX2* P2 promoter using MethylCap coupled with qPCR. Surprisingly, in contrast to the control groups, 5-mC levels remained unchanged in both the TET2-knockdown and the TET2-overexpressed groups (Figure 4, D and E).

To investigate whether the binding of TET2 modulates *RUNX2* gene transcription, we carried out a luciferase reporter assay driven by the *RUNX2* promoter using either the P1 or P2 promoter. Our results demonstrated that overexpression of TET2 significantly suppressed luciferase activity of the P2 promoter (Figure 4G), while having no effect on that of the P1 promoter (Figure 4F). In contrast to the WT TET2, an enzymatically inactive mutant form of TET2 exhibited a comparable inhibitory effect on the luciferase activity of the P2 promoter (Figure 4G). Moreover, we analyzed data from assays for transposase-accessible chromatin with high-throughput sequencing (ATAC-Seq) to investigate the chromatin accessibility of the *RUNX2* gene under different *TET2* interventions. We observed that, compared with the *TET2*-WT group, the *TET2*-KO group showed greater chromatin accessibility, as manifested by a markedly increased level of annotated peak in the *RUNX2* P2 promoter (Figure 4H). This indicates that TET2 KO led to the inhibition of the P2 promoter (GEO GSE241347). However, ATAC-Seq revealed no significant difference between the *TET2*-WT and the *TET2* loss-of-function mutation(*TET2*-MUT) groups (Figure 4I) (32), suggesting that the inhibitory role of TET2 at the P2 promoter is independent of its enzymatic function.

In order to examine the function of TET2 binding to the *RUNX2* promoter in the context of VC, we carried out CUT&Tag coupled with qPCR (CUT&Tag-qPCR, NovoNGS; CUT&Tag High-Sensitivity Kit, Cell Signaling Technology). An anti-TET2 antibody was used to immunoprecipitate protein-DNA complexes from both control and Pi-induced VSMCs. Subsequently, the *RUNX2* P1 (CUT1) and P2 (CUT2) promoters were amplified via qPCR (Figure 4J). The results indicated that TET2 was predominantly enriched on the P2 promoter and to a lesser extent on the P1 promoter. Notably, enrichment on the P2 promoter was significantly reduced during osteogenic transdifferentiation of hVSMCs induced by Pi (Figure 4J). Beyond their established roles in DNA oxidation, TET proteins have been reported to functionally interact with other epigenetic modifiers, thereby inducing chromatin remodeling and consequent gene transcription (33–35). In light of these findings, we performed CUT&Tag-qPCR by immunoprecipitating with antibodies that recognize the active marker of promoters, specifically the acetylation of lysine 27 on histone 3 (H3K27ac). As expected, we detected substantially elevated enrichment of H3K27ac at the *RUNX2* P2 promoter compared with the P1 promoter following Pi-induced hVSMC osteogenic transdifferentiation (Figure 4K), signifying establishment of an open chromatin conformation at the *RUNX2* P2 promoter. Compared with the control vector, knockdown of TET2 led to a significant increase in H3K27ac levels at the P2 promoter in hVSMCs (Figure 4L). Conversely, overexpression of TET2 markedly reduced H3K27ac levels at the P2 promoter, indicative of a repressive chromatin state (Figure 4M).

Collectively, the aforementioned results validated that TET2 directly suppresses *RUNX2* gene transcription by diminishing its H3K27ac levels on the P2 promoter, rather than through its DNA demethylation function.

### TET2 interacts with HDAC1/2 to suppress activity of the RUNX2 P2 promoter through deacetylation of H3K27ac.

**Figure 5:**
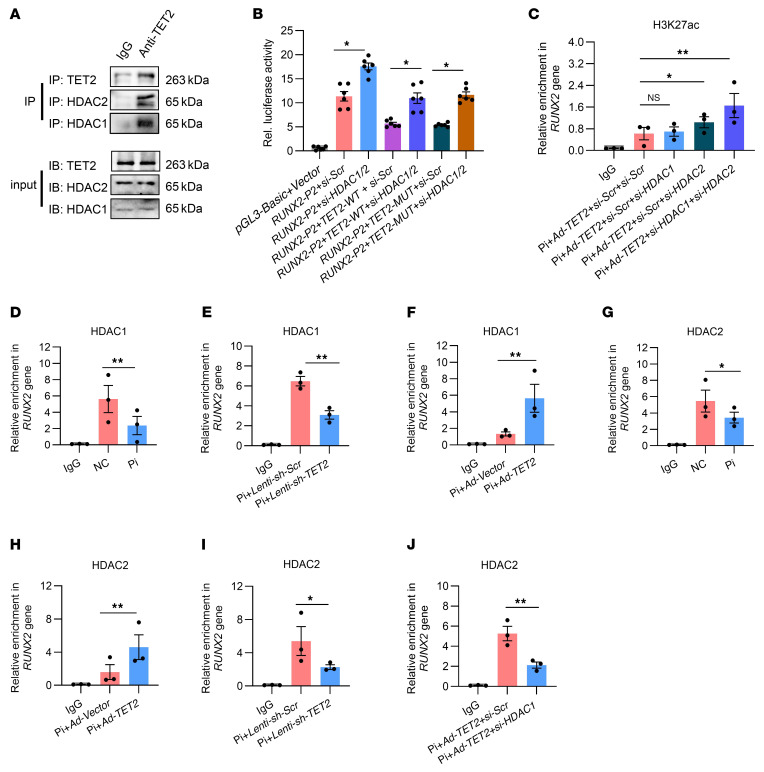
TET2 interacts with HDAC1/2 to suppress activity of the *RUNX2* P2 promoter through deacetylation of H3K27ac. (**A**) Co-IP analysis to detect the interaction between TET2 and HDAC1/2 in hVSMCs. (**B**) In VSMCs pretreated with control or HDAC1/2 knockdown, luciferase activities were analyzed after cotransfection with control Renilla luciferase plasmid and constructs of RUNX2 P2 promoter–driven luciferase reporters, and cotransfection with control, TET2-WT, or enzyme activity locus–mutated TET2 (*n* = 6 per group). (**C**) H3K27ac CUT&Tag-qPCR at the RUNX2 P2 promoter in hVSMCs transfected with *si-Scr*, *si-HDAC1*, *HDAC2*, or *HDAC1/2*, together with subjection to TET2 overexpression (*n* = 3 per group). (**D**–**I**) HDAC1 CUT&Tag-qPCR (**D**–**F**) or HDAC2 CUT&Tag-qPCR (**G**–**I**) at the *RUNX2* P2 promoter in hVSMCs with either Pi exposure (**D** and **G**), TET2 knockdown (**E** and **I**), or TET2 overexpression (**F** and **H**) (*n* = 3 per group). (**J**) HDAC2 CUT&Tag-qPCR at the *RUNX2* P2 promoter in TET2-overexpressing hVSMCs with either control or HDAC1 knockdown (*n* = 3 per group). All values are presented as mean ± SEM. *P < 0.05, **P < 0.01. Statistical significance was assessed using 1-way ANOVA followed by Dunnett’s test (**B**–**J**).

Previous evidence suggested that two histone deacetylases, HDAC1 and HDAC2, were associated with a majority of genes marked with H3K27ac and coexisted in several polyprotein repressive complexes that silenced genes (36). This suggested their potential roles in regulating VSMC osteogenic transdifferentiation in conjunction with TET2. First, we verified the endogenous interactions between TET2 and HDAC1/2 in hVSMCs (Figure 5A). Subsequently, we carried out a luciferase reporter assay driven by the *RUNX2* promoter using the P2 promoter. We discovered that knockdown of HDAC1/2 significantly reversed the inhibitory effect of TET2 on luciferase activity of the *RUNX2* P2 promoter. Similar reversed outcomes were also observed in the case of the enzymatically mutated form of TET2 (Figure 5B).

To explore whether HDAC1 and HDAC2 were involved in the regulation exerted by TET2 on *RUNX2*, we performed CUT&Tag-qPCR by immunoprecipitating H3K27ac in hVSMCs overexpressing TET2 and simultaneously transfected with *si-Scr*, *si-HDAC1*, *si-HDAC2*, or *si-HDAC1/2* separately. The results demonstrated that the enrichment of H3K27ac in the Runx2 P2 promoter increased slightly in the *si-HDAC2* group and showed no difference in the *si-HDAC1* groups, while significantly rising in the *si-HDAC1/2* group (Figure 5C). To further investigate the recruitment of HDAC1/2 to the *RUNX2* P2 promoter by TET2, we conducted CUT&Tag-qPCR using HDAC1 and HDAC2 antibodies separately. The results revealed that the binding of both HDAC1 (Figure 5D) and HDAC2 (Figure 5G) was dramatically decreased at the *RUNX2* P2 promoter after Pi stimulation. Enrichment of HDAC1 (Figure 5E) and HDAC2 (Figure 5I) at the P2 promoter declined in the TET2-knockdown groups, while substantially increasing after TET2 overexpression (Figure 5, F and H). Given that HDAC1 might compensate for the absence of HDAC2 at the *RUNX2* P2 promoter, we detected HDAC2 binding after knocking down HDAC1 and confirmed a marked reduction of HDAC2 at the P2 promoter (Figure 5J). These results imply that TET2 and HDAC1/2 form an inhibitory complex to inhibit the P2 promoter and consequently repress *RUNX2* gene transcription. In this complex, HDAC2 may serve as the key enzyme for deacetylating H3K27ac, while HDAC1 might act as a compensatory factor in the event of HDAC2 loss.

### TET2 inhibits VC by interacting with HDAC1/2.

**Figure 6:**
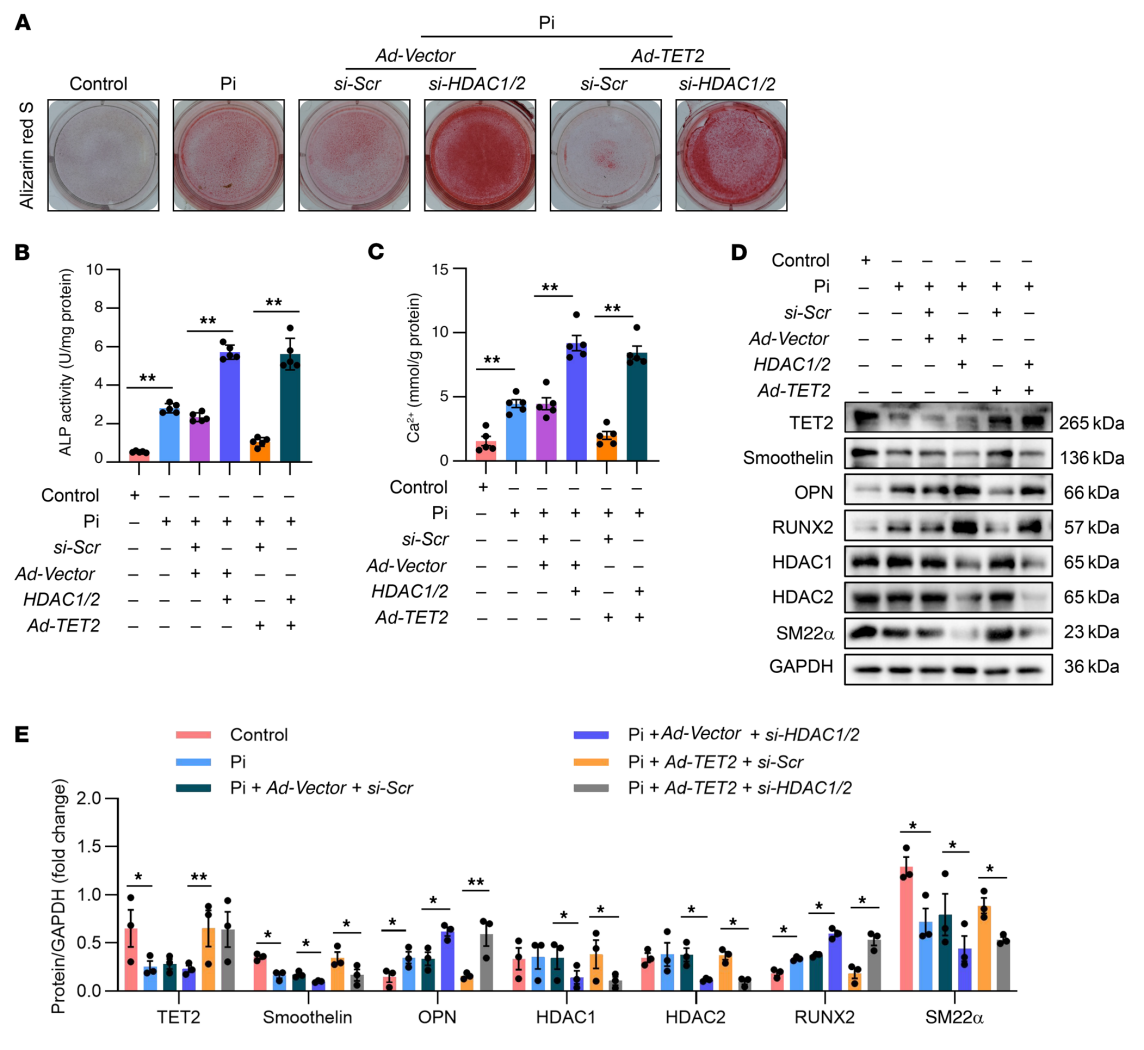
TET2 inhibits hVSMC osteogenic transdifferentiation by interacting with HDAC1/2. (**A**) Alizarin red staining of hVSMCs transfected with *si-Scr* or *si-HDAC1/2* together with *Ad-Vector* or *Ad-TET2* (*n* = 3 per group). (**B** and **C**) ALP activity assay (**B**) and quantification of calcium content (**C**) in hVSMCs transfected with *si-Scr* or *si-HDAC1/2* together with *Ad-Vector* or *Ad-TET2* (*n* = 5 per group). (**D** and **E**) Western blot analysis and quantification of TET2, RUNX2, OPN, smoothelin, SM22, and HDAC1/2 expression in hVSMCs transfected with *si-Scr* or *si-HDAC1/2* together with *Ad-Vector* or *Ad-TET2* (*n* = 3 per group). All values are presented as mean ± SEM. *P < 0.05, **P < 0.01. Statistical significance was assessed using 1-way ANOVA followed by Dunnett’s test (**B**, **C**, and **E**).

Subsequently, we delved into whether HDAC1/2 participated in the regulatory mechanism of TET2 in suppressing VC. By simultaneously knocking down HDAC1/2 and overexpressing TET2, we discovered that the knockdown of HDAC1/2 remarkably offset the protective effect of TET2 against hVSMC calcification. This was evidenced by alizarin red S staining (Figure 6A), quantification of ALP activity (Figure 6B), and calcium assay (Figure 6C). Moreover, further Western blot analysis demonstrated that, in contrast to the group with TET2 overexpression and *si-Scr* intervention, the group with TET2 overexpression and *si-HDAC1/2* intervention exhibited substantially greater expression of osteogenic differentiation genes, including OPN and RUNX2, while expressing lower levels of VSMC phenotype genes, including smoothelin and SM22α (Figure 6, D and E). Notably, the knockdown of HDAC1/2 was both highly efficient and specific (Supplemental Figure 3, C and D; and Supplemental Table 8).

### SNIP1 is necessary for TET2 to interact with HDAC1/2 at the RUNX2 P2 promoter.

**Figure 7:**
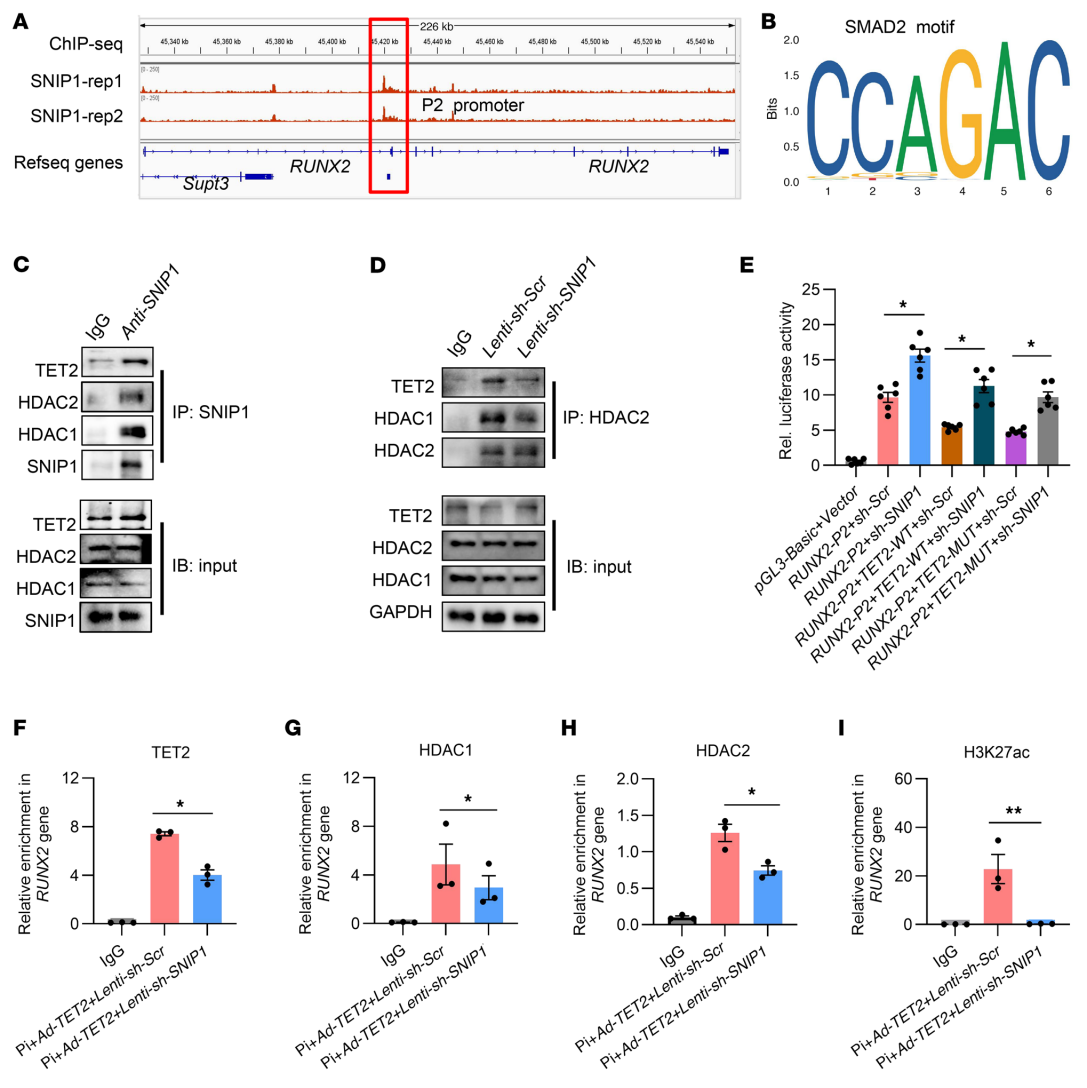
SNIP1 is necessary for TET2 to interact with HDAC1/2 at the *RUNX2* P2 promoter. (**A**) ChIP-Seq analysis for SNIP1 enrichments on the *RUNX2* gene. (**B**) SMAD2 binding motif. (**C**) Co-IP to detect the interaction between SNIP1, TET2, and HDAC1/2 in hVSMCs. (**D**) Co-IP analysis of hVSMCs pretransfected with Lenti-sh-Scr or Lenti-sh-SNIP1 to detect the interaction between TET2 and HDAC1/2. (**E**) Luciferase activity analysis of cells preinfected with Lenti-sh-Scr or Lenti-sh-SNIP1, after cotransfection with control Renilla luciferase plasmid and constructs of the P2 promoter–driven luciferase reporters; and cotransfection with control, TET2-WT, or enzyme activity locus–mutated TET2 (*n* = 6 per group). (**F**–**I**) TET2 (**F**), HDAC1 (**G**), HDAC2 (**H**), and H3K27ac (**I**) CUT&Tag-qPCR at the *RUNX2* P2 promoter in hVSMCs transfected with *Lenti-sh-Scr* or *Lenti-sh-SNIP1* together with TET2 overexpression (*n* = 3 per group). All values are presented as mean ± SEM. *P < 0.05, **P < 0.01. Statistical significance was assessed using 1-way ANOVA followed by Dunnett’s test (**E**–**I**).

TET2 lacks a CXXC DNA binding domain and is likely to bind to specific genes through other proteins, such as cell type–specific transcription factors (37–40). Previous findings have identified SMAD nuclear interacting protein 1 (SNIP1) as one of the potential transcription regulators that interact with TET2 (41, 42). SNIP1 is recognized as a transcription repressor that inhibits the BMP signaling pathway by directly interacting with its intracellular effectors, the SMAD2/3 proteins, thereby limiting its functions (43). There is evidence suggesting that SNIP1 inhibits the TGF-β/BMP signaling pathways by interfering with the interaction of SMAD2/3 and the histone acetyltransferase CBP/p300 (41). As is commonly known, the BMP signaling pathway is a key pathway in osteogenic differentiation, and its intracellular effectors, the SMAD2/3 proteins, are the key factors for the transcriptional activation of the *RUNX2* gene (6, 44–46). Taking the above evidence into account, we investigated whether SNIP1 is involved in the transcriptional inhibition exerted by the TET2-HDAC1/2 complex at the P2 promoter of the *RUNX2* gene. First, we analyzed ChiP-Seq data (47) on SNIP1-enriched chromatin and detected a significant peak of SNIP1 at the *RUNX2* P2 promoter (Figure 7A). Subsequently, we analyzed the binding motif within the ChIP-Seq peaks where TET2 and SNIP1 co-bind and found SMAD2 motifs to be present in the co-occupied peaks (Figure 7B). Furthermore, we identified the endogenous interactions between SNIP1 and TET2, and also found that SNIP1 interacted with HDAC1/2 in hVSMCs (Figure 7C). We hypothesized that SNIP1 might enhance the interaction between TET2 and HDAC1/2. Hence, we carried out co-IP experiments under the condition of SNIP1 knockdown. It was observed that in Lenti-sh-SNIP1 versus control VSMCs, the interaction between TET2 and HDAC1/2 was substantially reduced (Figure 7D). Then we performed a luciferase reporter assay driven by the *RUNX2* promoter specifically using the P2 promoter. We noticed that knockdown of SNIP1 significantly reversed the repressive effect of TET2 on the luciferase activity of the P2 promoter (Figure 7E), and similar reversed outcomes were seen in the enzymatic mutation form of TET2 (Figure 7E). To further investigate the in vivo interaction of SNIP1 and TET2 on the P2 promoter, we performed CUT&Tag-qPCR. We found that after SNIP1 knockdown, binding of TET2 at the P2 promoter significantly decreased (Figure 7F). Similarly, enrichment of HDAC1/2 also substantially declined (Figure 7, G and H), while H3K27ac was markedly increased (Figure 7I). Western blot data showed that the knockdown of SNIP1 was highly efficient (Supplemental Figure 3C). In conclusion, these findings led us to the conclusion that SNIP1 was essential for TET2 to interact with HDAC1/2 at the *RUNX2* P2 promoter and consequently for the removal of H3K27ac.

### SNIP1 is vital for TET2 to impede hVSMC osteogenic transdifferentiation.

**Figure 8:**
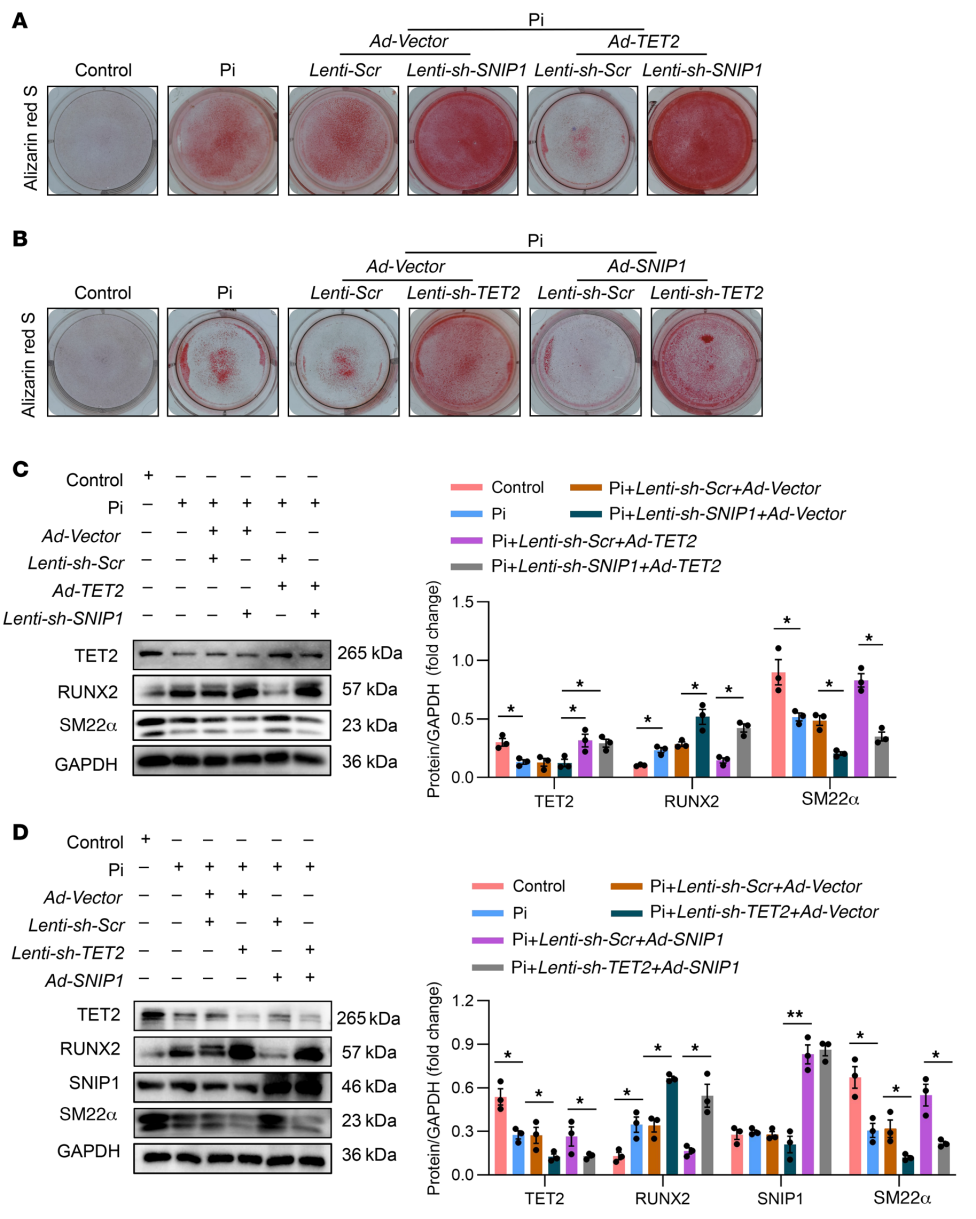
SNIP1 is vital for TET2 to hinder hVSMC osteogenic transdifferentiation. (**A** and **C**) Alizarin red staining (**A**) and Western blot analysis and quantification (**C**) of hVSMCs transfected with *Lenti-sh-Scr* or *Lenti-sh-SNIP1* together with *TET2*-overexpression or control vector (*n* = 3 per group). (**B** and **D**) Alizarin red staining (**B**) and Western blot analysis and quantification (**D**) of hVSMCs transfected with *Lenti-sh-Scr* or *Lenti-sh-TET2* together with *SNIP1* overexpression or control vector (*n* = 3 per group). All values are presented as mean ± SEM. *P < 0.05, **P < 0.01. Statistical significance was assessed using 1-way ANOVA followed by Dunnett’s test (**C** and **D**).

We proceeded to investigate the role of SNIP1 in VC. As anticipated, overexpression of SNIP1 in hVSMCs significantly alleviated VC and reduced RUNX2 expression (Supplemental Figure 4, A and B). To ascertain whether SNIP1 mediates osteogenic reprogramming of VSMCs regulated by TET2, we transfected hVSMCs with *Lenti-sh-SNIP1* in combination with *Ad-TET2*. We observed that loss of SNIP1 significantly attenuated the inhibitory effect of TET2 on hVSMC calcification and RUNX2 expression (Figure 8, A and C). Subsequently, hVSMCs were transfected with *Lenti-sh-TET2* along with *Ad-SNIP1*. Results confirmed that knockdown of TET2, in the context of SNIP1 overexpression, largely reversed the protective effects of SNIP1 on hVSMC calcification and RUNX2 expression (Figure 8, B and D). These findings therefore demonstrate that SNIP1 is essential for TET2 to inhibit VC.

### Knockdown of Snip1 accelerated VC in mice.

**Figure 9:**
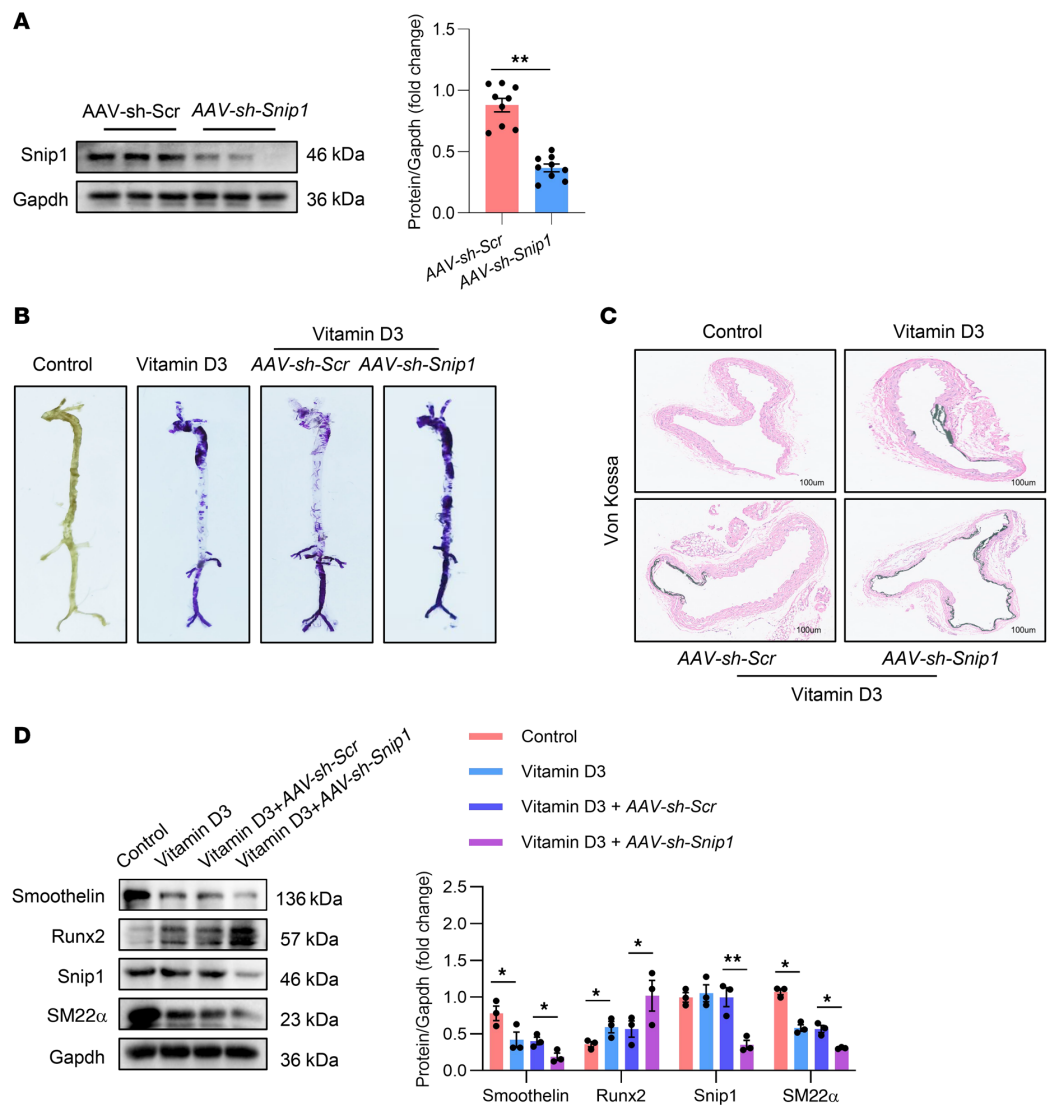
Knockdown of Snip1 accelerated VC in mice. (**A**) Western blot analysis and quantification of Snip1 expression in aortas from mice injected with *AAV-sh-Scr* and *AAV-sh-Snip1* (*n* = 3 per group). (**B**) Representative alizarin red S staining images of whole aortas from control mice, mice injected with vitamin D3, and mice injected with *AAV-sh-Scr* or *AAV-sh-Snip1* (*n* = 3 per group). (**C**) Representative von Kossa staining of aortic sections from control mice, mice injected with vitamin D3, and mice injected with *AAV-sh-Scr* or *AAV-sh-Snip1*. Scale bars: 100 μm. *n* = 3 per group. (**D**) Western blot analysis and quantification of Snip1, osteogenic phenotypic marker Runx2, and contractile phenotype marker (Smoothelin and SM22α) expression in the aortas from control mice, mice injected with vitamin D3, and mice injected with *AAV-sh-Scr* or *AAV-sh-Snip1* (*n* = 3 per group). All values are presented as mean ± SEM. *P < 0.05, **P < 0.01. Statistical significance was assessed using 2-tailed *t* tests (**A**) and 1-way ANOVA followed by Dunnett’s test (**D**).

To gain a more comprehensive understanding of the role of SNIP1 in VC, we employed adeno-associated virus (AAV) infection as a genomic manipulation model. AAVs with *TAGLN* promoter carrying either scrambled shRNA or *Snip1* shRNA (*sh-Snip1*) were administered via the tail vein in vitamin D3–induced mouse models. Depletion of Snip1 in the aorta was verified through Western blot analysis (Figure 9A; sequences of si-Snip1 are listed in Supplemental Table 8). Loss of Snip1 remarkably augmented calcium deposition and mineralization in the aorta when compared with scrambled control, as confirmed by alizarin red S staining (Figure 9B). Consistent with this finding, von Kossa staining demonstrated substantially increased calcium deposition in aortic sections of Snip1-knockdown mice (Figure 9C). Further Western blot analysis indicated significantly increased expression of osteogenic genes Runx2, while markedly decreased expression of VSMC genes including smoothelin and SM22α (Figure 9D). Taken together, these results indicate that a deficiency of Snip1 accelerates VC.

## Discussion

In this work, we identified TET2 as an inhibitor of VSMC osteogenic transdifferentiation, which functions directly to repress the transcription of *RUNX2* gene. Through a comprehensive series of in vitro and in vivo experiments, we have established a crucial role of TET2 in inhibiting VSMC calcification. Notably, TET2 expression is downregulated in calcified samples from both humans and mouse models. Experiments involving TET2 deletion and overexpression established a causal role between TET2 and VSMC calcification, such that TET2 deficiency exacerbates VC, whereas TET2 overexpression leads to a significant attenuation of VC. In addition, application of such experiments using human arteries from the same vascular sites, with and without calcification, will further support our findings.

Previous evidence has suggested a close correlation between TET2 and cardiovascular disease (24, 25, 48), as well as its significance for the normal differentiation of VSMCs (28). However, the effects of TET2 on VC still remain a mystery. In this study, we revealed the critical role of TET2 in VC and discovered that RUNX2 may be the target of TET2 in protecting VSMCs from VC. As we have known, VSMCs have plastic ability and are able to differentiate into other cell types in response to environmental changes (9, 49), and VSMC osteogenic transdifferentiation is one of the results. Previous results show that TET2 is a master regulator of the VSMC contractile phenotype, altering DNA methylation to promote expression of MYOCD, SRF, and other contractile genes (28). Also, coordinate suppression of *KLF4* and other dedifferentiation-related genes has been discovered in VSMCs (28), but what directs TET2’s mediation of the opposing effects on contractile and dedifferentiated genes is still unclear. Now we have confirmed that TET2 is essential and necessary for VSMC osteogenic transdifferentiation. We conclude that the absence of TET2 in VSMCs results in repression of VSMC contractile genes but activation of *RUNX2* gene transcription. Ectopic expression of TET2 in VSMCs not only promoted expression of VSMC contractile genes but also contributed to repression of osteogenic genes. Taken together, these results may offer clues as to why VSMCs have the ability to transdifferentiate into osteogenic cells instead of other phenotypes, and the loss of TET2 in VSMCs may be the key culprit.

Mechanistically, we found that TET2 can bind to the *RUNX2* gene P2 promoter and repress its activity, and the enzymatic loss-of-function mutation had the same effect. Independent of its DNA demethylation function, we found that TET2 facilitates HDAC1/2 binding to the *RUNX2* P2 promoter, which led to histone deacetylation–mediated inhibition of *RUNX2*. Moreover, SNIP1 is necessary for TET2 to interact with HDAC1/2 at the *RUNX2* P2 promoter and is vital for TET2 to hinder VC. Furthermore, most regulators of *RUNX2* transcription reported to date concentrate on its P1 promoter (the remote promoter) (29, 50, 51). Here, we provide the evidence that TET2 correlates with *RUNX2* transcription inhibition by binding to the special locus of its P2 promoter (the proximal promoter).

Compelling evidence regarding TET proteins to date have concentrated on their DNA demethylation function (22, 28, 48), but the functions of TET2 and the effects of its enzymic loss mutations in VC are largely unknown. In this study, we show that, independent of the DNA demethylation roles, TET2 coordinated with HDAC1/2 to inhibit *RUNX2* gene transcription, and the enzymatic mutations had the same effect. Previous studies have revealed that, except for the known regulatory roles in DNA demethylation, TET proteins are able to coordinate with other epigenetic modifiers to induce multilayer chromatin regulation. For example, it has been reported that TET2 can connect with H3K4 methylation to upregulate gene transcription (33, 35). TET1 has also been revealed to participate in the silencing of developmental genes in embryonic stem cells (52). Recently, the repressing roles of TET2 and its nonenzymatic function have begun to be understood. In line with our study, a previous study revealed that TET2 can repress gene transcription in chromatin not by its catalytic activity, but by interacting with histone deacetylase complexes (36). Another study also suggested that PSPC1 and TET2 can act together with histone deacetylase complexes for transcriptional silencing of MERVL and this occurs independently of TET2 catalytic activity (53). Studies also show that IFN signaling was restrained by TET2 in human macrophages, and DNA methylation lacks correlation with the activation of IFN signaling. The authors found that TET2 interacts with RBPJ and ZNF143 in regulatory regions of the transcription factor A mitochondria (*TFAM*) gene to regulate the expression of the *TFAM* gene (54). Moreover, our study is also consistent with reports that revealed that loss of catalytic roles of TET2 are crucial to homeostasis in hematopoietic stem and progenitor cells (55).

Increasing evidence has revealed that most chromatin-modifying enzymes are not bound to the target DNA by themselves (56), but are recruited to specific genes by other factors to regulate their expression and cellular processes. In this study, we discovered the critical role of TET2 in *RUNX2* gene transcription. TET2, however, is unable to bind to target genes by themselves (37, 38), and previous studies have reported the coregulating roles of SNIP1 in TET2 regulation (42). Evidence suggested that SNIP1 inhibits the TGF-β/BMP signaling pathways by interfering with the interaction of SMAD2/3 and the histone acetyltransferase CBP/p300 (41). As is commonly known, the BMP signaling pathway is a key pathway in osteogenic differentiation, and its intracellular effectors, the SMAD2/3 proteins, are the key factors for the transcriptional activation of the RUNX2 gene (44–46). We therefore investigated the roles of SNIP1 in *RUNX2* gene transcription and VSMC osteogenic transdifferentiation. We discovered that SNIP1 can bind to the *RUNX2* P2 promoter, and we analyzed the binding motif in TET2- and SNIP1-co-bound ChIP-Seq peaks, which revealed the presence of SMAD2 motifs in the co-occupied peaks. Further, we found that SNIP1 is necessary for TET2 to interact with HDAC1/2 at the *RUNX2* P2 promoter and is vital for TET2 to hinder VC. Our findings are concordant with previous work showing that SNIP1 is a transcription repressor that inhibits the BMP signaling pathway, limiting its effects by interacting directly with SMAD2/3 proteins (43). Evidence also suggests that SNIP1 inhibits the TGF-β/BMP signaling pathway by interfering with the interaction of SMAD2/3 with the histone acetyltransferase CBP/p300 (41).

Collectively, the current research endeavor has elucidated the pivotal role played by TET2 in safeguarding VSMCs against VC. It has furnished what we believe to be a novel mechanism explicating how the deficiency of TET2 within VSMCs triggers their dedifferentiation process and subsequent transdifferentiation specifically into osteogenic cells rather than alternative cell phenotypes. Moreover, we have revealed the function of the TET2-HDAC1/2-SNIP1 complex in the transcriptional regulation of the *RUNX2* gene. This finding imparts perspectives into the biochemical mechanism by which TET2 exerts its inhibitory effect on gene transcription, thereby enhancing our comprehension of the molecular underpinnings governing VSMC fate determination and the pathophysiological processes associated with VC.

## Methods

Further information can be found in Supplemental Methods.

### Sex as a biological variable.

Sex was not considered as a biological variable in human samples, and no difference was found between the sexes. Our study performed experiments on male and female mice, with similar findings reported for both sexes.

### Human samples.

Human arteries were collected from patients with CKD undergoing arterial venous fistula operation and diagnosed with aortic arch calcification (CKD, *n* = 6). Additionally, control arteries were obtained from patients who underwent amputation surgery due to upper-limb trauma, without a diagnosis of CKD or diabetes mellitus (control, *n* = 6). Blood samples were collected from CKD patients with calcification (*n* = 24) and without calcification (*n* = 12) and from healthy people (*n* = 21). Histopaque-1077 (MilliporeSigma) gradients were used to extract PBMCs from blood. For the CKD patients from whom we collected blood samples, we also collected their clinical and biochemical parameters from the electronic medical system. from the electronic medical system in the hospital. Related clinical samples were collected at Donghua Hospital of Sun Yat-sen University from November 2019 to January 2020.

### Animal experiments.

We performed experiments on male and female mice. Eight-week-old C57BL/6J mice were purchased from the Laboratory Animal Center of Sun Yat-sen University. To build VSCM-specific TET2-knockdown mice, we first constructed recombinant AAV9 gene transfer vectors carrying the *TAGLN* promoter and *sh-TET2* or *sh-Scr*, which we injected into the lateral tail vein of mice. The *sh-TET2* sequence is provided in Supplemental Table 7. After 4 weeks, we sacrificed 6 mice with isoflurane (induction 5%, maintenance 2%) and collected the aortas to verify the efficiency of *AAV-sh-TET2* in aortas. Then, to induce arterial medial calcification, we randomly injected mice with vitamin D3 (5.5 × 10^5^ U/kg/d) 3 days as previous described (57). About 6–8 days later, we sacrificed the mice and collected whole aortas for the following experiments. For the adenine diet–induced CKD model, mice were randomly provided a chow diet as the control group or a special diet containing 0.2% adenine and 1.2% phosphorus as the CKD group. Four weeks subsequent to the commencement of the specialized diet regime, the mice were administered the specified virus (at a dosage of 5 × 10^9^ PFU per kilogram body weight per mouse) via the tail vein injection method. After a lapse of 4 weeks, the mice were subjected to overnight fasting. Their body weights were recorded prior to euthanasia, and blood samples were collected. Subsequently, entire aortas were harvested and meticulously dissected for further in-depth analyses.

### Alizarin red staining.

Alizarin red staining was performed to determine hVSMC and mouse aorta calcification. First, cells were washed with PBS, then fixed with 4% paraformaldehyde. Next, they were washed with distilled water. Finally, we add 1% alizarin red solution and incubated the cells for 15 minutes, then washed them with distilled water. Mouse aortas were fixed with 4% paraformaldehyde for 24 hours, stained with 0.003% alizarin red solution in 1% sodium hydroxide for 30 hours, then washed with 1% sodium hydroxide. Positive results presented as a reddish color, indicating calcification.

### Von Kossa staining.

Slides of mouse aorta were deparaffinized and rehydrated, incubated in 5% silver nitrate, and exposed to ultraviolet light for about 1 hour to stain the calcified area brown or black. Finally, the slides were treated with 5% sodium thiosulfate and washed twice with double-distilled water. The calcified areas are stained brown or black.

### Calcium and ALP quantification.

For calcium quantification, the VSMCs were washed gently with PBS 3 times, then incubated the with 0.6 mol/L HCl overnight at 4°C. We then collected the supernatant. We used a commercial kit (Biosino Bio–Technology and Science) to measure calcium content according to the manufacturer’s instructions. For ALP quantification, we incubated the VSMCs with 1% Triton X-100 in 0.9% saline on ice, then collected the supernatant and performed centrifugation in a microfuge at 8000*g* for 5 minutes. We then used the assay kit to analyze ALP activity. Results were normalized by total protein levels.

### Laboratory analyses.

Mouse blood levels of blood urea nitrogen (BUN) and creatinine (CREA) were measured by an autoanalyzer (Hitachi). Plasma levels of calcium were measured using the detection kit (Biosino Bio-Technology and Science). Plasma levels of ALP were measured using a commercial assay kit (Biosino Bio-Technology and Science). Plasma levels of ALT and AST were analyzed using ELISA kits (Jiangsu Meimian Industrial) according to the manufacturer’s instructions.

### Co-IP.

We placed 25µL Pierce Protein A/G Magnetic Beads into a 1.5mL microcentrifuge tube, added 175µL of wash buffer to the beads, and gently vortexed to mix. We removed and discarded the supernatant. We then added 1mL of wash buffer to the tube, inverted the tube several times, and again removed and discarded the supernatant. We then added the antigen sample/antibody mixture and incubated at room temperature for 1 hour. We collected the beads and repeated the wash twice. Then add purified water wash once. Finally, we added Low-pH Elution Buffer and incubated for 10 minutes. We added Neutralization Buffer to neutralize the low pH, then boiled with SDS buffer and analyzed by Western blot. Antibodies are listed in Supplemental Table 4.

### Reverse transcription and qPCR.

Total RNA was extracted from peripheral leukocytes or cultured cells by TRIzol Reagent (Takara 9109) and reverse transcribed into cDNA with a Prime ScriptRT Reagent Kit (Takara RR036A). qPCR was performed using Bio-Rad SYBR Green on a CFX96 Touch Real-Time PCR Detection System (Bio-Rad). GAPDH was used as a reference and was calculated according to the 2^–ΔΔCt^ method. Primer sequences are listed in Supplemental Table 5.

### Immunohistochemical staining.

The sections were heated at 60°C for 1 hour and deparaffinized and rehydrated. 0.3% H_2_O_2_ was used to block endogenous peroxidase activity for 20 minutes. 10% citrate buffer and heat were used for antigen retrieval. Then primary antibodies were incubated overnight at 4°C, followed by a EnVision+ Dual Link System-HRP for 1 hour at room temperature. Finally, a DAB peroxidase substrate kit (ZSGB Bio, 2L2-9018) was used to stain the sections for 1 minute. Images were captured with light microscopy (Nikon NiU).

### Data analysis.

Normalization of gene counts and identification of related genes were performed by using DESeq2. The software enriched domain detector was used to detect wide genomic enriched domains. Using enriched domain detector, we calculated the TET2, SNIP1, and ATAC-enriched signal compared with input. Bigwig files were generated by the log_2_ ratio fold-change against input and visualized using the Integrative Genomics Viewer.

### Statistics.

GraphPad Prism 9.0 software was used to analyze all data. Values are presented as mean ± SEM. Two-tailed Student’s *t* test or nonparametric Mann-Whitney *U* test were performed to compare 2 groups. 1-way ANOVA followed by post hoc Bonferroni’s or Dunnett’s test was performed to compare multiple groups. Pearson’s correlation coefficient analysis was used to value the statistical correlations. *P* values less than 0.05 were considered significant.

### Study approval.

The collection of human arteries and blood samples from patients were approved by all donors enrolled in this study. This study was conducted in accordance with the Declaration of Helsinki and was approved by the Internal Review and Ethics Committee of the Donghua Hospital of Sun Yat-sen University (SYSEC-KY-KS-2020-191). The experimental animal protocols were approved by the Ethics Committee of Shenzhen TopBiotech Co. (TOP-IACUC-2023-0198).

### Data availability.

Data for bulk RNA-Seq analysis were from the GEO database (GSE159832 and GSE254077). TET2 and SNIP1 ChIP-Seq raw data and ATAC-Seq raw data used for analyses were from the GEO database (GSM7996293, GSE175848, GSE241347 and GSE213768). The data supporting the findings of this study are included in the main article, supplemental materials, and Supporting Data Values file.

## Author contributions

DH and HH designed the research. DH and JM performed most of the experiments. ZZ and HZ performed the animal experiments and analyzed the data. YQ, YL, and FW performed some of the biochemical and biophysical experiments. DH and HH wrote the manuscript with comments from all authors. HH, TZ, and HY revised the manuscript. TZ, HZ, and HY financially supported this investigation. All authors approved the final version of this manuscript. We determined the order of co–first authors by their efforts and contributions to this project.

## Supplementary Material

Supplemental data

Unedited blot and gel images

Supporting data values

## Version 1 03/11/2025

In-Press Preview

## Version 2 05/01/2025

Electronic publication

## References

[1] Schlieper G (2008). Vascular access calcification predicts mortality in hemodialysis patients. Kidney Int.

[2] Zhang W (2023). Impaired intracellular calcium homeostasis enhances protein O-GlcNAcylation and promotes vascular calcification and stiffness in diabetes. Redox Biol.

[3] Timmis A (2018). European Society of Cardiology: Cardiovascular Disease Statistics 2017. Eur Heart J.

[4] Lanzer P (2014). Medial vascular calcification revisited: review and perspectives. Eur Heart J.

[5] Shanahan CM (2013). Mechanisms of vascular calcification in CKD-evidence for premature ageing?. Nat Rev Nephrol.

[6] Ouyang L (2021). ALKBH1-demethylated DNA N6-methyladenine modification triggers vascular calcification via osteogenic reprogramming in chronic kidney disease. J Clin Invest.

[7] Li W (2022). SIRT6 protects vascular smooth muscle cells from osteogenic transdifferentiation via Runx2 in chronic kidney disease. J Clin Invest.

[8] Furmanik M (2020). Reactive oxygen-forming Nox5 links vascular smooth muscle cell phenotypic switching and extracellular vesicle-mediated vascular calcification. Circ Res.

[9] Alexander MR, Owens GK (2012). Epigenetic control of smooth muscle cell differentiation and phenotypic switching in vascular development and disease. Annu Rev Physiol.

[10] Lacolley P (2017). Vascular smooth muscle cells and arterial stiffening: relevance in development, aging, and disease. Physiol Rev.

[11] Petsophonsakul P (2022). Nicotine promotes vascular calcification via intracellular Ca2+-mediated, Nox5-induced oxidative stress, and extracellular vesicle release in vascular smooth muscle cells. Cardiovasc Res.

[12] Chen Y (2020). Arterial stiffness: a focus on vascular calcification and its link to bone mineralization. Arterioscler Thromb Vasc Biol.

[13] Chen Y (2021). Transcriptional programming in arteriosclerotic disease: a multifaceted function of the Runx2 (Runt-related transcription factor 2). Arterioscler Thromb Vasc Biol.

[14] Byon CH (2008). Oxidative stress induces vascular calcification through modulation of the osteogenic transcription factor Runx2 by AKT signaling. J Biol Chem.

[15] Shao JS (2006). Molecular mechanisms of vascular calcification: lessons learned from the aorta. Arterioscler Thromb Vasc Biol.

[16] Steitz SA (2001). Smooth muscle cell phenotypic transition associated with calcification: upregulation of Cbfa1 and downregulation of smooth muscle lineage markers. Circ Res.

[17] Engelse MA (2001). Vascular calcification: expression patterns of the osteoblast-specific gene core binding factor alpha-1 and the protective factor matrix gla protein in human atherogenesis. Cardiovasc Res.

[18] Tyson KL (2003). Osteo/chondrocytic transcription factors and their target genes exhibit distinct patterns of expression in human arterial calcification. Arterioscler Thromb Vasc Biol.

[19] Sun Y (2012). Smooth muscle cell-specific runx2 deficiency inhibits vascular calcification. Circ Res.

[20] Heath JM (2014). Activation of AKT by O-linked N-acetylglucosamine induces vascular calcification in diabetes mellitus. Circ Res.

[21] Lin ME (2015). Runx2 expression in smooth muscle cells is required for arterial medial calcification in mice. Am J Pathol.

[22] Ito S (2010). Role of Tet proteins in 5mC to 5hmC conversion, ES-cell self-renewal and inner cell mass specification. Nature.

[23] Potus F (2020). Novel mutations and decreased expression of the epigenetic regulator *TET2* in pulmonary arterial hypertension. Circulation.

[24] Soubrier F (2020). TET2: a bridge between DNA methylation and vascular inflammation. Circulation.

[25] Gumuser ED (2023). Clonal hematopoiesis of indeterminate potential predicts adverse outcomes in patients with atherosclerotic cardiovascular disease. J Am Coll Cardiol.

[26] Kuhnert S (2022). Association of clonal hematopoiesis of indeterminate potential with inflammatory gene expression in patients with COPD. Cells.

[27] Abplanalp WT (2020). Association of clonal hematopoiesis of indeterminate potential with inflammatory gene expression in patients with severe degenerative aortic valve stenosis or chronic postischemic heart failure. JAMA Cardiol.

[28] Liu R (2013). Ten-eleven translocation-2 (TET2) is a master regulator of smooth muscle cell plasticity. Circulation.

[29] Drissi H (2000). Transcriptional autoregulation of the bone related CBFA1/RUNX2 gene. J Cell Physiol.

[30] Harada H (1999). Cbfa1 isoforms exert functional differences in osteoblast differentiation. J Biol Chem.

[31] Zeng Q (2024). A negative feedback loop between TET2 and leptin in adipocyte regulates body weight. Nat Commun.

[32] Huerga Encabo H (2023). Loss of TET2 in human hematopoietic stem cells alters the development and function of neutrophils. Cell Stem Cell.

[33] Williams K (2011). TET1 and hydroxymethylcytosine in transcription and DNA methylation fidelity. Nature.

[34] Chen Q (2013). TET2 promotes histone O-GlcNAcylation during gene transcription. Nature.

[35] Deplus R (2013). TET2 and TET3 regulate GlcNAcylation and H3K4 methylation through OGT and SET1/COMPASS. EMBO J.

[36] Zhang Q (2015). Tet2 is required to resolve inflammation by recruiting Hdac2 to specifically repress IL-6. Nature.

[37] Iyer LM (2009). Prediction of novel families of enzymes involved in oxidative and other complex modifications of bases in nucleic acids. Cell Cycle.

[38] Ko M (2013). Modulation of TET2 expression and 5-methylcytosine oxidation by the CXXC domain protein IDAX. Nature.

[39] Ko M (2010). Impaired hydroxylation of 5-methylcytosine in myeloid cancers with mutant TET2. Nature.

[40] Tahiliani M (2009). Conversion of 5-methylcytosine to 5-hydroxymethylcytosine in mammalian DNA by MLL partner TET1. Science.

[41] Kim RH (2000). A novel smad nuclear interacting protein, SNIP1, suppresses p300-dependent TGF-beta signal transduction. Genes Dev.

[42] Chen LL (2018). SNIP1 recruits TET2 to regulate c-MYC target genes and cellular DNA damage response. Cell Rep.

[43] Chng Z (2010). SIP1 mediates cell-fate decisions between neuroectoderm and mesendoderm in human pluripotent stem cells. Cell Stem Cell.

[44] Wu M (2024). The roles and regulatory mechanisms of TGF-β and BMP signaling in bone and cartilage development, homeostasis and disease. Cell Res.

[45] Salazar VS (2016). BMP signalling in skeletal development, disease and repair. Nat Rev Endocrinol.

[46] Watson KE (1994). TGF-beta 1 and 25-hydroxycholesterol stimulate osteoblast-like vascular cells to calcify. J Clin Invest.

[47] Cui H (2022). The SWI/SNF chromatin remodeling factor DPF3 regulates metastasis of ccRCC by modulating TGF-β signaling. Nat Commun.

[48] Ostriker AC (2021). TET2 protects against vascular smooth muscle cell apoptosis and intimal thickening in transplant vasculopathy. Circulation.

[49] Owens GK (2004). Molecular regulation of vascular smooth muscle cell differentiation in development and disease. Physiol Rev.

[50] Lee MH (2005). Dlx5 specifically regulates Runx2 type II expression by binding to homeodomain-response elements in the Runx2 distal promoter. J Biol Chem.

[51] Zambotti A (2002). Characterization of an osteoblast-specific enhancer element in the CBFA1 gene. J Biol Chem.

[52] Wu H (2011). Dual functions of Tet1 in transcriptional regulation in mouse embryonic stem cells. Nature.

[53] Guallar D (2018). RNA-dependent chromatin targeting of TET2 for endogenous retrovirus control in pluripotent stem cells. Nat Genet.

[54] Cobo I (2022). DNA methyltransferase 3 alpha and TET methylcytosine dioxygenase 2 restrain mitochondrial DNA-mediated interferon signaling in macrophages. Immunity.

[55] Ito K (2019). Non-catalytic roles of Tet2 are essential to regulate hematopoietic stem and progenitor cell homeostasis. Cell Rep.

[56] Smith E, Shilatifard A (2010). The chromatin signaling pathway: diverse mechanisms of recruitment of histone-modifying enzymes and varied biological outcomes. Mol Cell.

[57] Zeng P (2021). ERK1/2 inhibition reduces vascular calcification by activating miR-126-3p-DKK1/LRP6 pathway. Theranostics.

