# Supplementary material for: TET2 suppresses vascular calcification by forming an inhibitory complex with HDAC1/2 and SNIP1 independent of demethylation
Source: J Clin Invest. 2025 Mar 11;135(9):e186673. doi: 10.1172/JCI186673 (PMC12043087; doi:10.1172/JCI186673)
Supplement: Supplemental data [file jci-135-186673-s126.pdf]

SUPPLEMENTAL MATERIAL

Methods

CUT&Tag -qPCR.

hVSMC were harvested. 1× 10 5 cells were counted and centrifuged for 3 min at 600×g at room temperature, and washed twice in wash buffer. Following the manufacture, Concanavalin A coated magnetic beads were prepared, 10μL of activated beads were added each sample and incubated for 10 min at RT. Then, Cells were orderly incubated with ConA Beads, primary antibody, secondary antibody (Goat anti-Rabbit IgG H&L, AB206-01-AA) and Hyperactive PG-TN5 / PA-TN5 Transposon and then fragmented. The fragmented DNA was extracted from the samples and amplified by PCR. qPCR was performed to confirm the enrichment. Related primers are listed in Supplemental Table 5.

Methylated-DNA capture (MethylCap)- qPCR.

Genomic DNA was extracted from hVSMC by a QIAamp DNA Mini kit (QIAGEN, Cat#51304). MethylCap kit (Diagenode) was used to capture the methylated DNA. In brief, first we diluted the genomic DNA to reach 0.1μg/μl and sheared to 200 – 500bp. Next, incubate the genomic DNA with a His6-GST-MBD protein (the methyl-binding domain (MBD) of the human MeCP2 protein fused with a glutathione-S-transferase (GST) and containing an N-terminal His6-tag) coupled to magnetic beads. Then wash the beads and eluted DNA. Finally, qPCR was performed with the methylated DNA and input fractions to confirm enrichment of the methylated genes in each group. Related primers are listed in Supplemental Table 5.

Plasmid construction and luciferase reporter assay.

The serial DNA fragments from the human RUNX*2* P2 promoter and P1 promoter were amplified by PCR. The PCR products were cloned into pGL3-Vector to generate the RUNX*2* luciferase reporter constructs. Wild type and enzymatic activity mute type TET2 over expressed plasmids were constructed. Sequencing was performed to verify the constructs. After the appropriated treatment, the reporter plasmid and internal control Renilla luciferase plasmids were co-transfected with into hVSMC by electroporation. Luciferase activity was detected by the Dual-Luciferase Reporter Assay Kit (Promega) according to the manufacturer's instructions. Primer sequences can be found in Supplemental Table 5.

Immunofluorescence.

Slides were deparaffinized, rehydrated, and antigen retrieval. Then normal goat serum in PBS/0.1% Triton X-100 to block the slides. Then primary antibody for mouse anti– α-SMA (Abcam), rabbit anti-TET2 (Proteintech) were incubated overnight at 4°C. Alexa Fluor (Jackson ImmunoResearch) secondary antibodies incubate at 37°C for 1 hour at room temperature. DAPI were used for Nuclei counterstain. Images were pictured by Olympus fluorescence microscope.

Western blot analysis.

Cells or tissue were added with radio immunoprecipitation assay (RIPA) lysis buffer and protease inhibitor PMSF on ice for 30 minutes. Collected the lysate

by centrifuge at 12,000 rpm at 4ºC for 20 minutes. Then quantify the supernatant by BCA assay kit (Thermo scientific, Cat#23252). Proteins were separated in SDS-polyacrylamide gel electrophoresis and transferred to PVDF membrane (Merck Millipore, Burlington, MA). Then block the membranes and then incubated overnight at 4 °C with primary antibodies. Then incubated with anti HRP-conjugated antibody (diluted 1:10,000) for 1 hour at room temperature. ClarityTM Western ECL Substrate (Bio-rad, Cat#170- 5061) were used to expose them.

Cell culture and VSMCs calcification induction*.*

hVSMC were purchased from the American Type Culture Collection and cultured in high-glucose Dulbecco’s modified Eagle’s medium (DMEM) containing 10% fetal bovine serum (FBS; Gibco), 100 U/ mL penicillin, and 100 µg/mL streptomycin in a 37°C incubator with 5% CO2. To induce VC, an additional 2.8 mmol/L of sodium phosphate (Pi) (Sigma) was added into the DMEM containing 10% fetal bovine serum for 7 days, and refreshed once every 2 days.

Cells were transfected with indicated viruses or siRNA respectively and then incubated for one night prone to infection. For control small interfering RNA (siRNA), Lipofectmaine RNAiMAX according to the manufacturer’s instructions (Invitrogen, 13778150). The sequences of si-RNA and sh-RNA are listed in Supplemental Table 6 and 8.

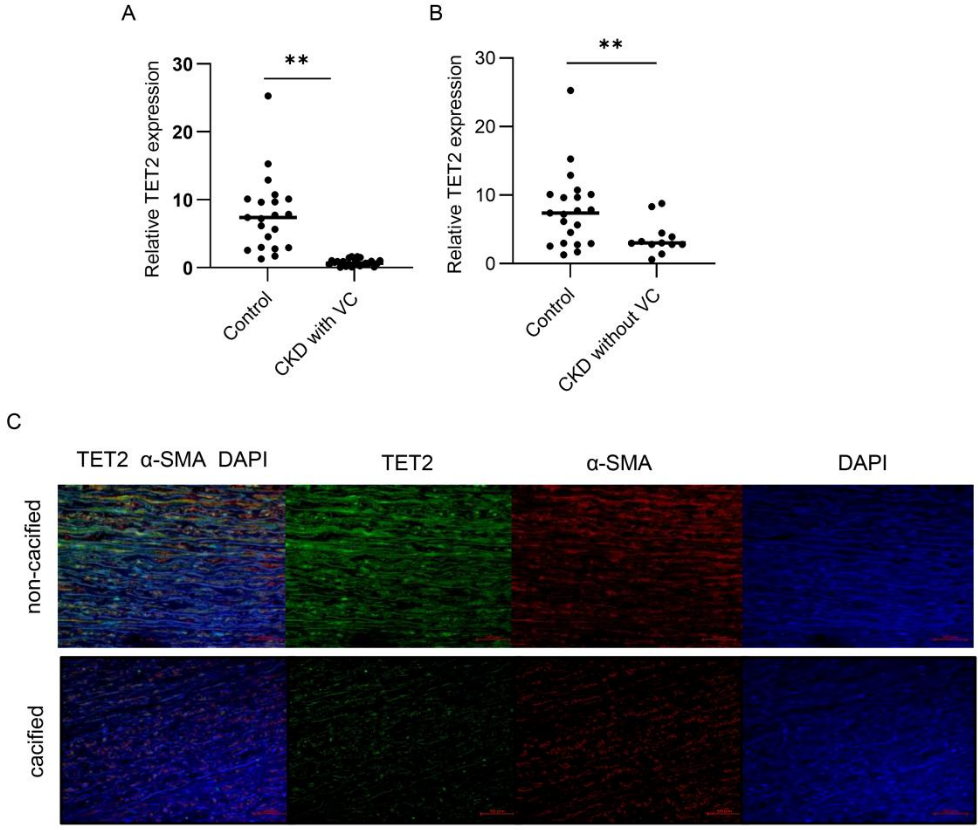

Supplemental Figure 1: TET2 expression decreased both in CKD patients and calcified human species. (A) Leukocyte TET2 expression in healthy people and CKD patients with VC (n = 21). (B) Leukocyte TET2 expression in healthy people and CKD patients without VC (n = 12). (C) Representative immunofluorescence pictures in calcified and non-calcified arteries from patients with CKD, Scale bars: 50μm, n=6. Data are presented as mean ± SEM, **P<0.01, *P<0.05.

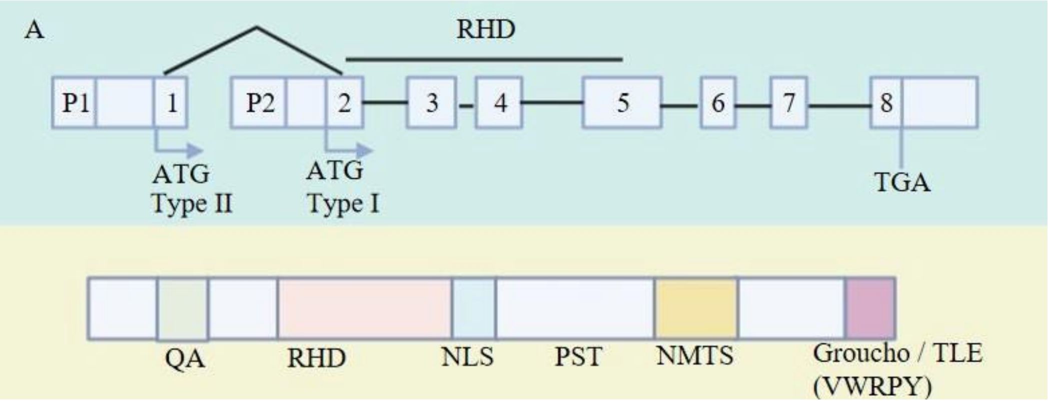

Supplemental Figure 2：*RUNX2* gene structure. *RUNX2* is transcriptionally regulated by two promoters. the distal P1 promoter and the proximal P2 promoter. Two major RUNX2 isoforms are transcribed from the P1 and P2 promoters respectively, indicating by the ATG start codons, which are encoded by exon 1–8 (type II) or exons 2–8 (type I

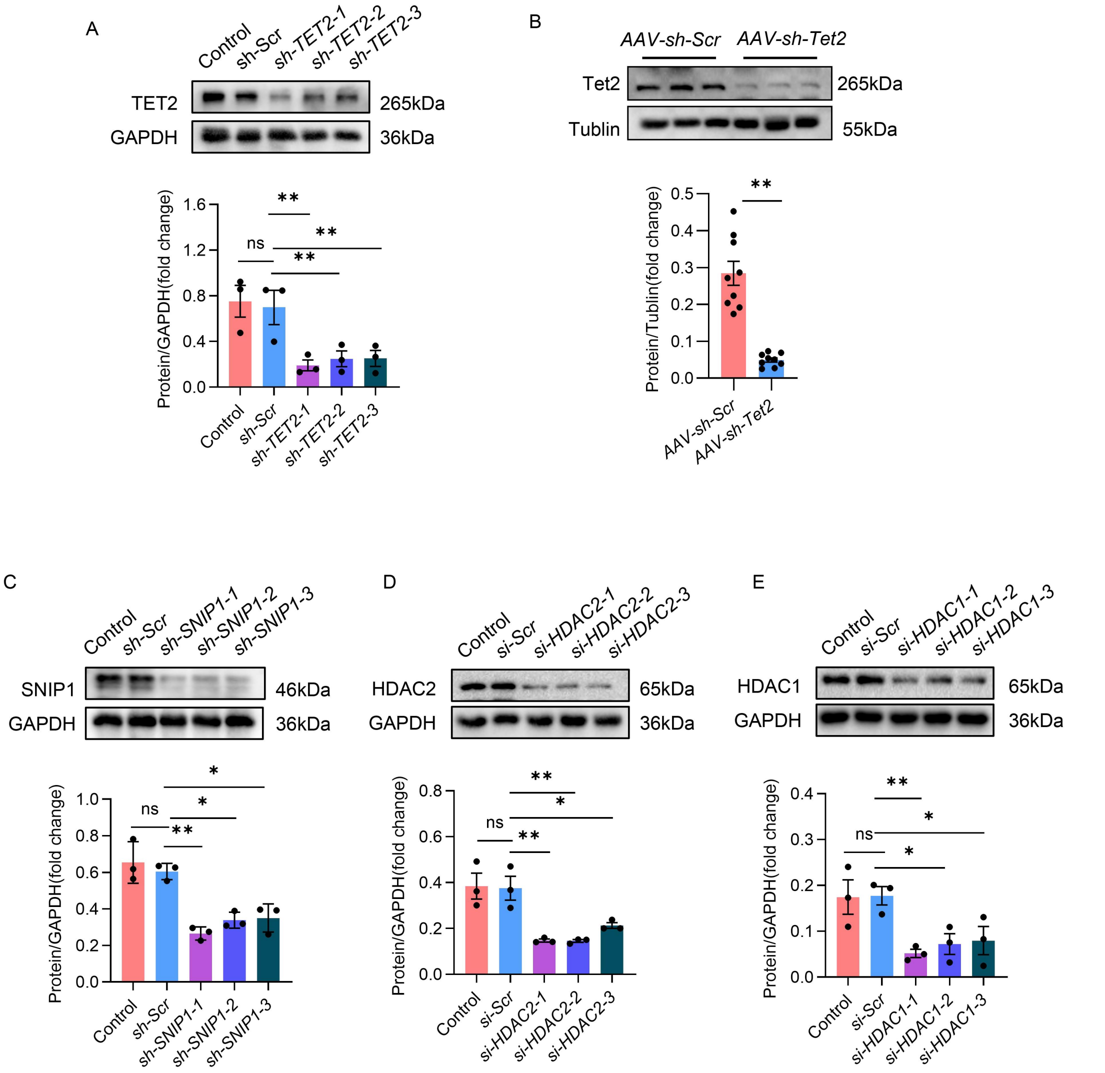

Supplemental Figure 3: knockdown efficiency of related sh-RNA or si-RNA.(A) Western blot analysis of TET2 expression respectively with either a control, sh-Scr, sh-1, sh-2, sh-3 (n = 3 per group). (B) Western blot analysis and quantification of Tet2 expression in mice aortas for *AAV-sh-Scr* and *AAV-sh-Tet2* (n = 5 per group). (C-D) Western blot analysis of SNIP1, HDAC1, HDAC2 expression respectively with either a control, si- or sh-Scr, si- or sh-1, si- or sh-2, si- or sh-3 (n = 3 per group); Data are presented as mean ± SEM, **P<0.01, *P<0.05.

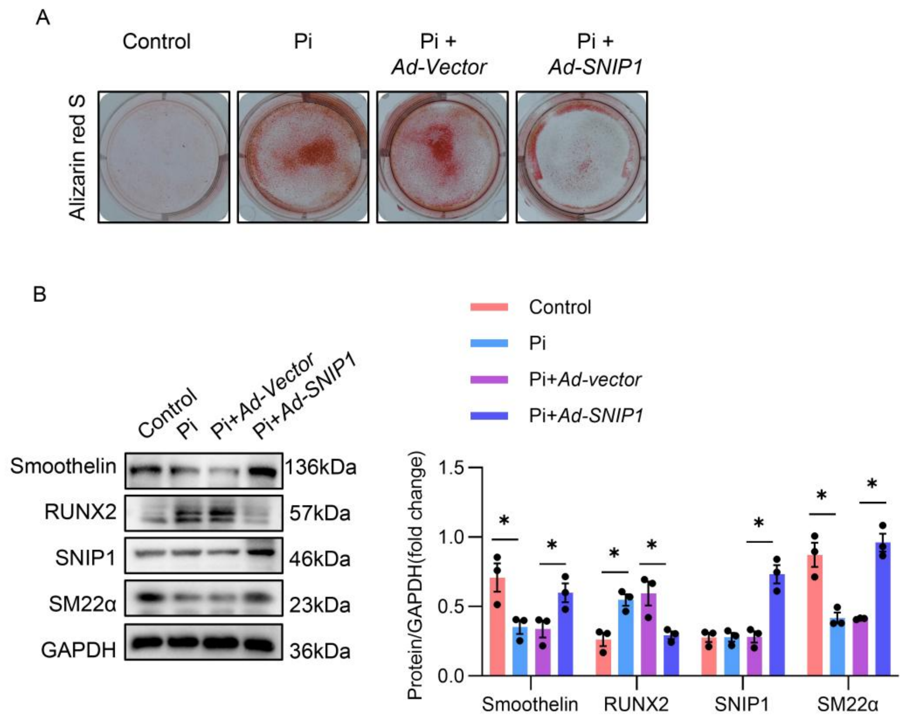

Supplemental Figure 4: overexpression of SNIP1 inhibits vascular calcification. (A) Alizarin red staining of in hVSMC of control, Pi, transfected with *Ad-Vector*, and *Ad-SNIP1* (n = 3 per group); (B) Western blot analysis of Smoothelin, RUNX2, SNIP1, SM22α expression in hVSMC of control, Pi, transfected with *Ad-Vector*, and *Ad-SNIP1* (n = 3 per group). Data are presented as mean ± SEM, **P<0.01, *P<0.05. Statistical significance was assessed using one-way ANOVA followed by Dunnett’s test (B).

Supplemental Table 1: Baseline characteristics of CKD patients with or without vascular calcification .

Characteristics	Calcified (n = 24)	Non-calcified (n = 12)	P value
SEX (male)	12 (50%)	4 (33.3%)	0.343
Age, years	61.33 ± 9.26	62.83 ± 10.26	0.661
BMI, kg/m²	24.89 ± 4.22	22.02 ± 2.09	0.068
SBP, mmHg	134.87 ± 18.68	121.83 ± 17.75	0.078
DBP, mmHg	75.87 ± 10.25	77.50 ± 8.94	0.667
BUN, mmol/l	6.05 (5.45, 7.02)	5.80 (5.10, 6.90)	0.579
Serum CR, mmol/l	471.9 (114.8, 1135.2)	513.3 (111.4, 786.3)	0.663
TC, mmol/L	1.30 (1.09,1.58)	1.08 (0.82, 2.68)	0.763
TG, mmol/L	1.83 ± 1.57	1.36 ± 0.40	0.309
HDL-C, mmol/L	1.16 ± 0.39	1.20 ± 0.19	0.799
LDL-C, mmol/L	3.01 ± 0.97	2.96 ± 0.85	0.876

Continuous values are expressed as mean ± SD or median (25th to 75th quartiles) for and categorical variables are *n (*%), respectively. Student's *t* test (all characteristics except sex) or nonparametric Mann Whitney *U* test (sex) were used to access statistical significance. BMI, body mass index; SBP, systolic blood pressure; DBP, diastolic blood pressure; BUN, Blood urea nitrogen; CR, creatinine; HDL, high-density lipoprotein; LDL, low-density lipoprotein.

Supplemental Table 2. Baseline characteristics of vitamin D3 mice model.

**Characteristics**	**Control**	**Vitamin D3**	**VitaminD3+ *AAV-sh-Scr***	**VitaminD3+ *AAV-sh-TET2***
Weight (g)	27.74±0.61	16.29±1.09*	16.04±1.13	16.32±0.53
ALP (U/L)	18.79±1.70	42.29±5.24*	40.64±5.25	94.40±19.44#
Serum Ca (mmol/l)	1.38±0.11	1.41±0.10	1.38±0.13	1.38±0.22
Serum BUN (mg/dl)	18.89±1.55	18.92±1.24	19.05±1.63	18.86±1.48
Serum Cr (umol/l)	23.51±3.12	24.94±2.66	24.78±3.17	25.59±2.63
ALT (pg/ml)	30.54±2.41	29.37±2.95	29.43±1.47	30.30±6.04
AST (pg/ml)	56.09±3.48	55.38±4.16	55.36±4.46	55.7±8.07

All values are presented as mean ± SD. The symbol "*" represents that compared to the control group P <0.05, and the symbol "#" represents a P <0.05 when compared to the CKD + sh-Scr intervention group. Statistical significance was assessed using Student's t test.Ca, calcium; BUN, blood urea nitrogen; Cr, creatinine; ALT, alanine transaminase; AST, aspartate aminotransferase; ALP, alkaline phosphatase;

Supplemental Table 3. Baseline characteristics of adenine-diet-induced CKD mice model.

**Characteristics**	**Control**	**CKD**	**CKD+*AAV-sh-Scr***	**CKD+*AAV-sh-TET2***
Weight (g)	28.84±0.76	18.56±1.01*	17.97±1.19	18.22±0.98
ALP (U/L)	22.22±3.65	46.08±5.55*	45.50±5.15	105.56±17.95#
Serum Ca (mmol/l)	1.44±0.20	1.38±0.19	1.46±0.21	1.55±0.17
Serum BUN (mg/dl)	18.70±1.29	46.68±1.82*	47.22±2.16	46.93±1.53
Serum Cr (umol/l)	27.59±3.17	89.76±9.15*	89.67±9.00	89.77±10.71
ALT (pg/ml)	29.86±4.68	30.31±4.08	29.65±4.60	30.95±3.77
AST (pg/ml)	56.79±3.48	56.79±7.70	56.80±6.01	56.45±2.71

All values are presented as mean ± SD. The symbol "*" represents that compared to the control group P <0.05, and the symbol "#" represents a P <0.05 when compared to the CKD + sh-Scr intervention group. Statistical significance was assessed using Student's t test.Ca, calcium; BUN, blood urea nitrogen; Cr, creatinine; ALT, alanine transaminase; AST, aspartate aminotransferase; ALP, alkaline phosphatase;

Supplemental Table 4: The antibodies used in this study

**Antibodies**		
TET2	CST #18950S	WB 1:1000; IP: 1:200; IF: 1:200; Chromatin IP: 1:50
TET2	Protein tech #21207-1-AP	WB 1:1000; IP: 1:200; IHC: 1:200;
SNIP1	Abcam #ab19611	WB 1:1000; IP: 1:200; Chromatin IP: 1:50
HDAC1	Protein tech #16160-1-AP	WB 1:1000; IP: 1:200; Chromatin IP: 1:50
HDAC2	Protein tech #16152-1-AP	WB 1:1000; IP: 1:200; Chromatin IP: 1:50
RUNX2	Abcam #ab76956	WB 1:1000; IHC:1:200
RUNX2	CST #12556s	IP: 1:200; IF: 1:200; Chromatin IP: 1:50
Smoothelin	Proteintech #23567-1-AP	WB 1:1000
SM22	Abcam # ab14106	WB 1:1000
OPN	Protein tech #22952-1-AP	WB 1:1000
GAPDH	Protein tech # 60004-1-Ig	WB 1:1000
Tublin	Protein tech #11224-1-AP	WB 1:1000
Anti-rabbit- FITC	Sigma # F9887	IF: 1:2000
Anti-mouse-Alexa Flu-647	Abcam # ab150115	IF: 1:2000
Anti-rabbit	CWBIO # CW0103	WB 1:10000
Anti-mouse	CWBIO # CW0102	WB 1:10000
IgG	CST #2729	IP: 2μl for 1mg protein

Supplemental Table 5: Primers for qPCR

Primer	Forward Sequence	Reverse Sequence
hRUNX2	5'-TgTTAATgCCATAgCTCCTTgCTg-3'	5'-AgCCAAgAAggAAATgACACATCg-3'
hTET2	5'-TTCgCAgAAgCAgCAgTgAAgAg-3'	5'-AgCCAgAgACAgCgggATTCCTT-3'
hGAPDH	5'-ACAACTTTggTATCgTggAAgg-3'	5'-gCCATCACgCCACAgTTTC-3'
RUNX2 (human) primers for CUT&Tag assay and MethylCap		
P2 promoter (CpG1)	5'-ggACTTgAgTTTgCAgCTTggAAT-3'	5'-CTgAACCCACACCgCTTCAC-3'
P2 promoter (CpG2)	5'-CTTCAAggTgCCAAgAggTAAgTC-3'	5'-CTAggCTTCggAAACTgCACAC-3'
P1 promoter	5'-CTTAACCTTACAggAgTTTgggCTC -3'	5'-AgCACTATTACTggAgAggCAgAAT -3'
RUNX2 (human) promoter luciferase constructs		
P2 promoter	GATCGCCGTGTAATTCTAGACGCGACC<br>TGGCGAGATTGCC	CCGGCCGCCCCGACTCTAGACGGGG<br>CGAGGCGCAGAGCTCGGTC
P1 promoter	TTTCTCTATCGATAGGTACCAAACCAT<br>GTTTGGAAATCCC	CTTAGATCGCAGATCTCGAGTTAAAAA<br>CCAGAGACTTCTTGCTG

Supplemental Table 6: The Relative sh-RNA in this work

NO.	5’	STEM	Loop	STEM	3’
(human)*SNIP1-RNAi-a*	Ccgg	CCAGTCATGTACATACATCGA	CTCGAG	TCGATGTATGTACATGACTGG	TTTTTg
(human)*SNIP1-RNAi-b*	aattcaaaaa	CCAGTCATGTACATACATCGA	CTCGAG	TCGATGTATGTACATGACTGG	
(human)*TET2-RNAi-a*	Ccgg	ATGCCAGTAAACTAGCTGCAA	CTCGAG	TTGCAGCTAGTTTACTGGCAT	TTTTTg
(human)*TET2-RNAi-b*	aattcaaaaa	ATGCCAGTAAACTAGCTGCAA	CTCGAG	TTGCAGCTAGTTTACTGGCAT	

Supplemental Table 7: The Relative AAV-shRNA in this work

AAV-shTet2-RNAi	gacgagctgtacaagg<br>ctagc<br>TAACTGGAGGC<br>TTGC<br>TGAAGGCTGTA<br>TGCT G	TTGGTGT<br>T<br>CAAATTC<br>AA CCTG	GTTTT<br>GG<br>CCACT<br>GA<br>CTGAC	CAGGTT<br>GA<br>TTGAAC<br>AC CAA	CAGGACACAAGG<br>CCTG<br>TTACTAGCACTCA<br>CATG<br>GAACAAATGGCC<br>Caagc<br>tttaaatagctgaggcc

Supplemental Table 8: The Relative si-RNA in this work

(human) *si-HDAC1*	GCUUCAAUCUAACUAUCAAdTdT	UUGAUAGUUAGAUUGAAGCdTdT
(human) *si-HDAC2*	GGAAGAAGAUAAAUCCAAGTT	CUUGGAUUUAUCUUCUUCCTT
(mouse) *si-Snip1*	UAUUCUCUGCUACUGAACCTT	GGUUCAGUAGCAGAGAAUATT
