# Supplementary material for: TET2 suppresses vascular calcification by forming an inhibitory complex with HDAC1/2 and SNIP1 independent of demethylation
Source: J Clin Invest. 2025 Mar 11;135(9):e186673. doi: 10.1172/JCI186673 (PMC12043087; doi:10.1172/JCI186673)

Full unedited gel for Figure 1H

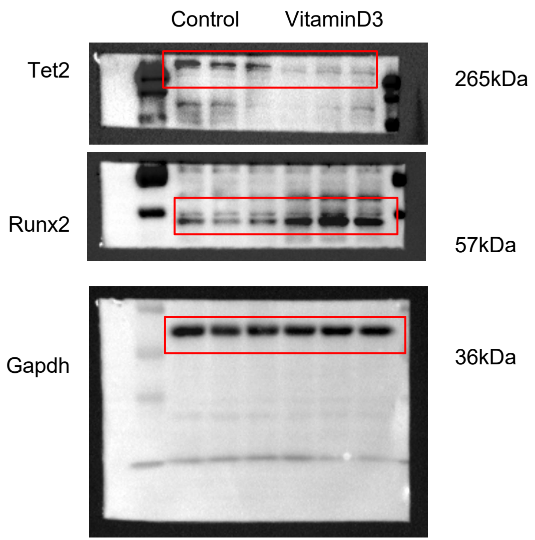

Full unedited gel for Figure 1I

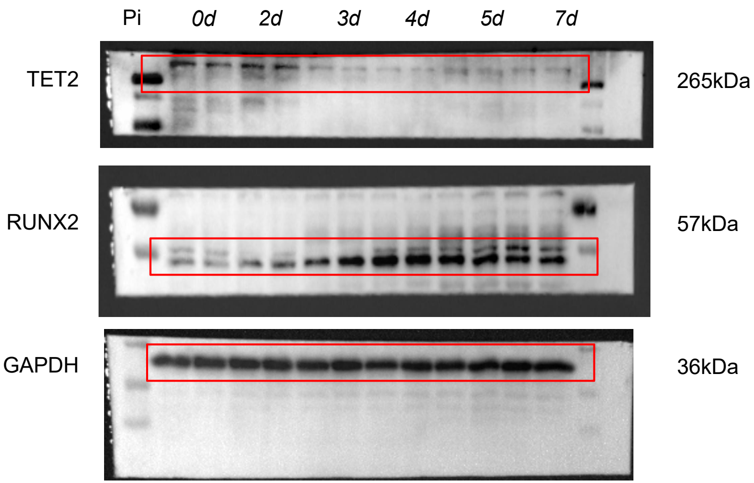

Full unedited gel for Figure 2G

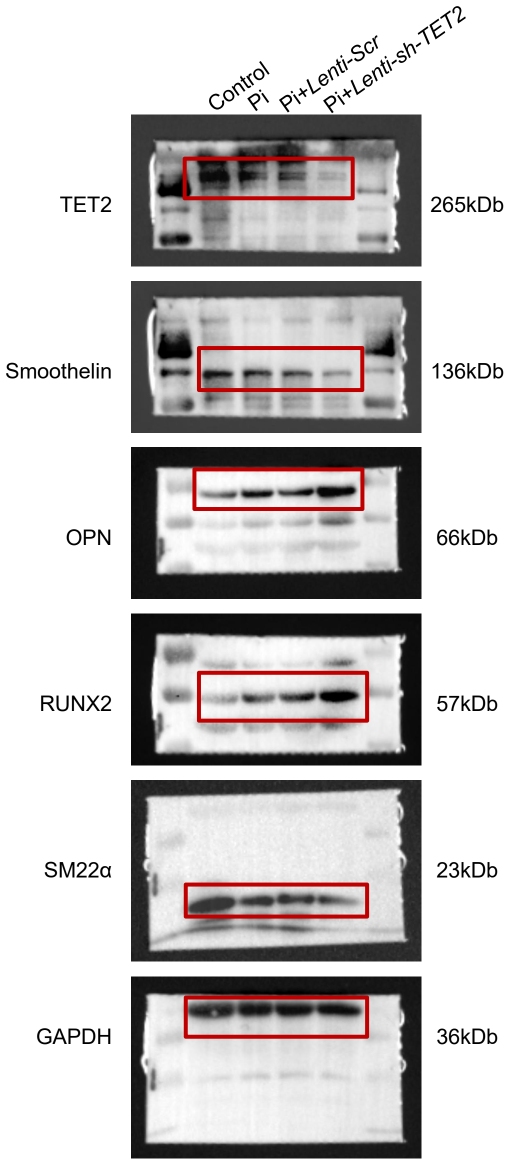

Full unedited gel for Figure 2H

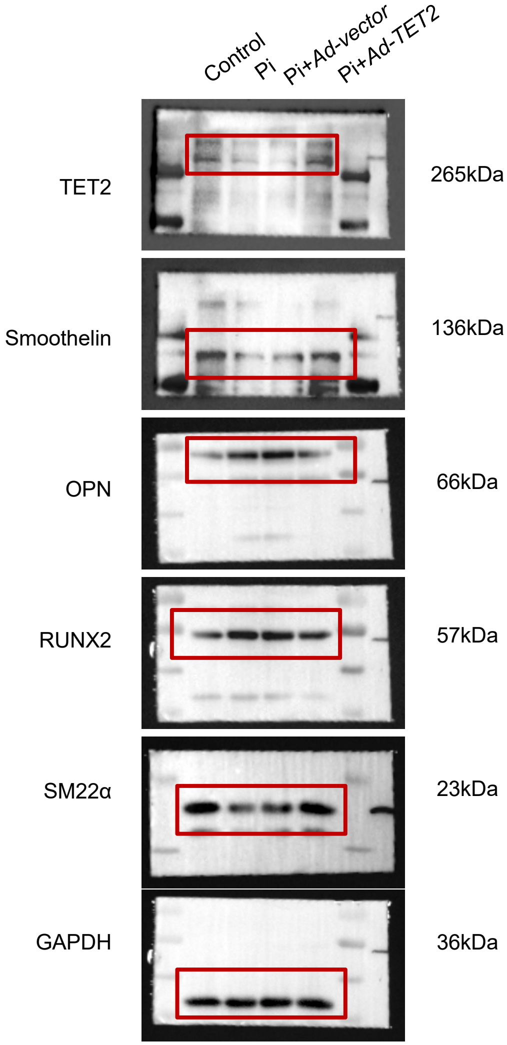

Full unedited gel for Figure 3D

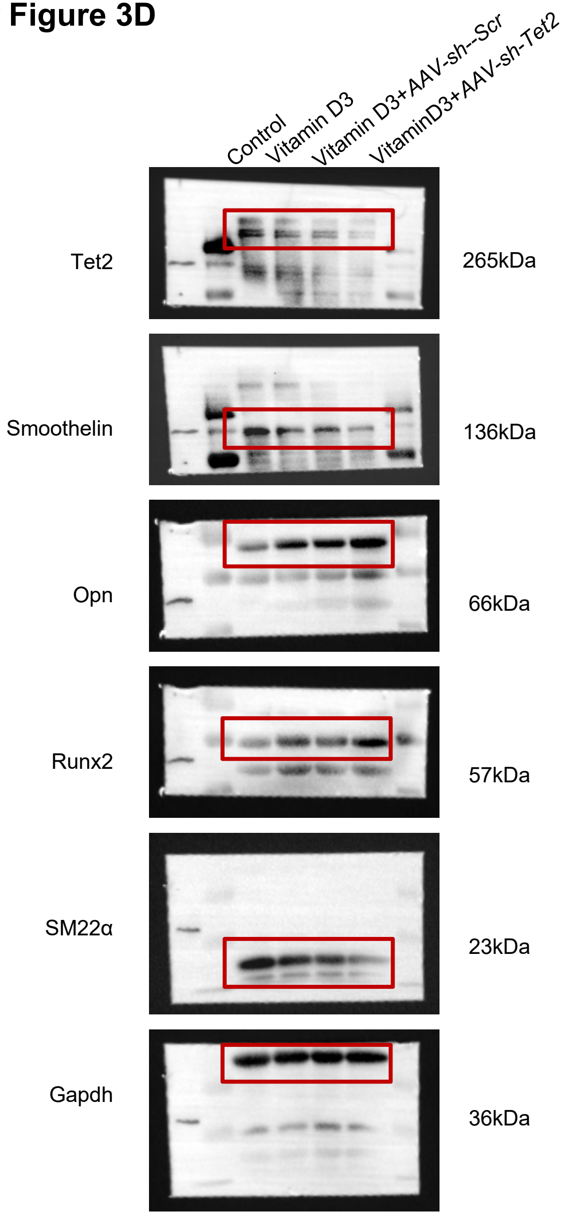

Full unedited gel for Figure 3G

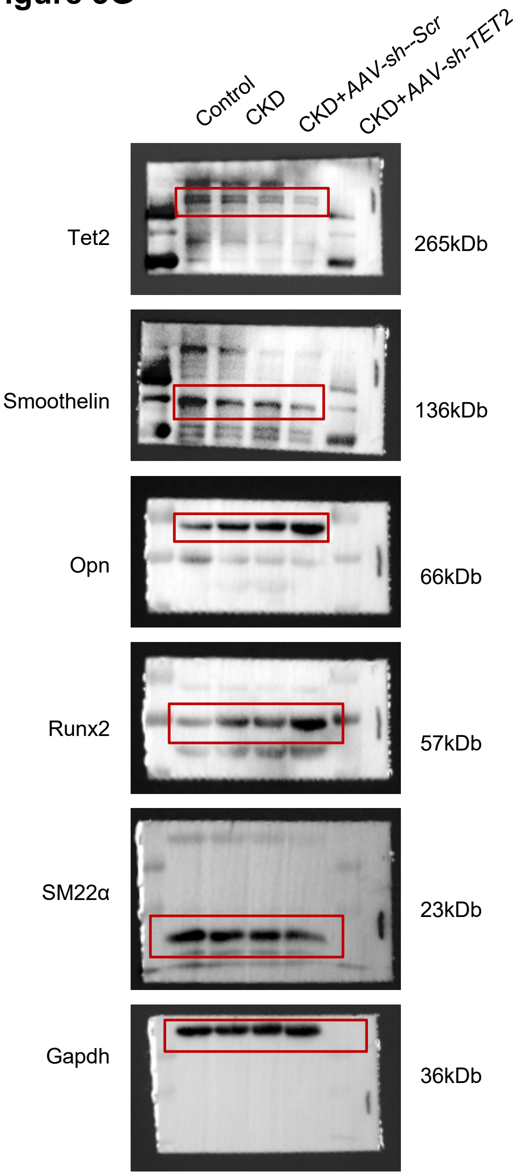

Full unedited gel for Figure 5A

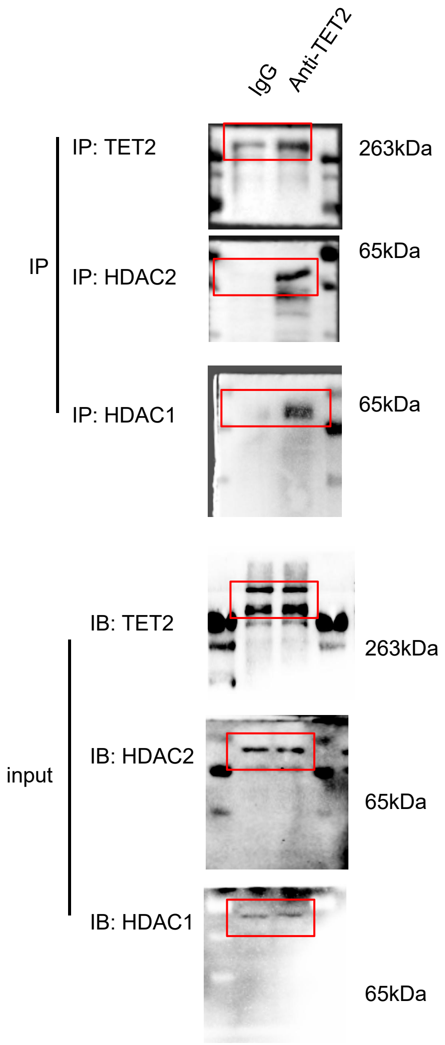

Full unedited gel for Figure 6D

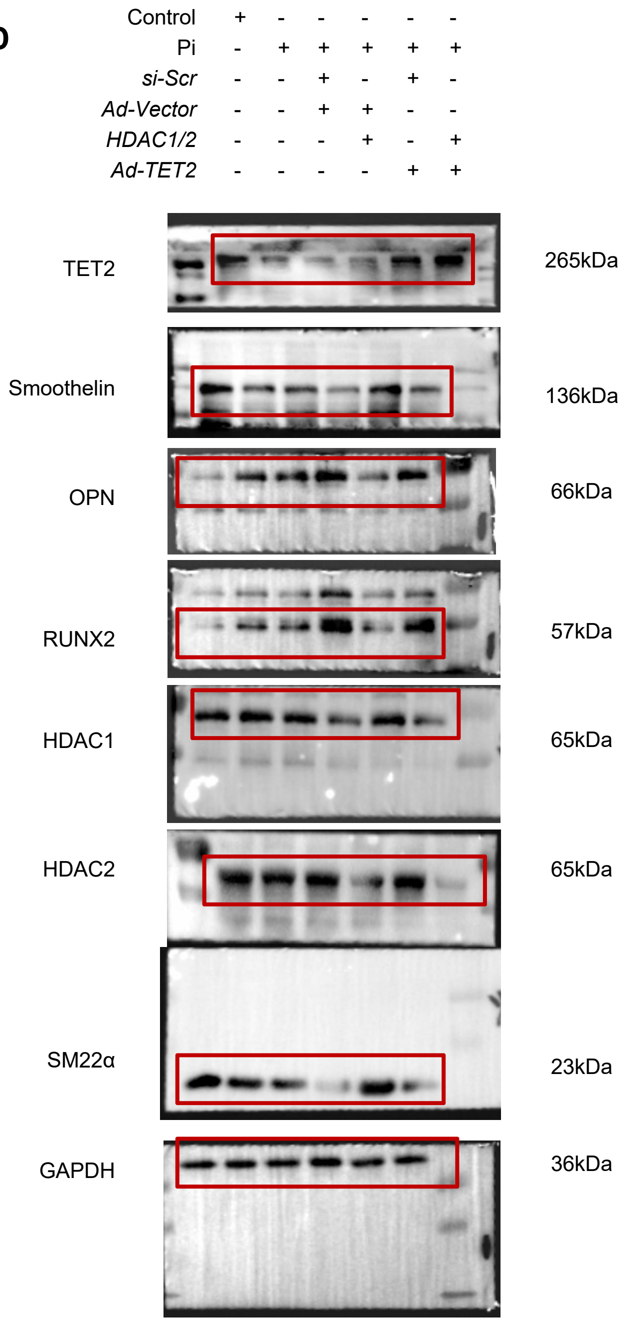

Full unedited gel for Figure 7C

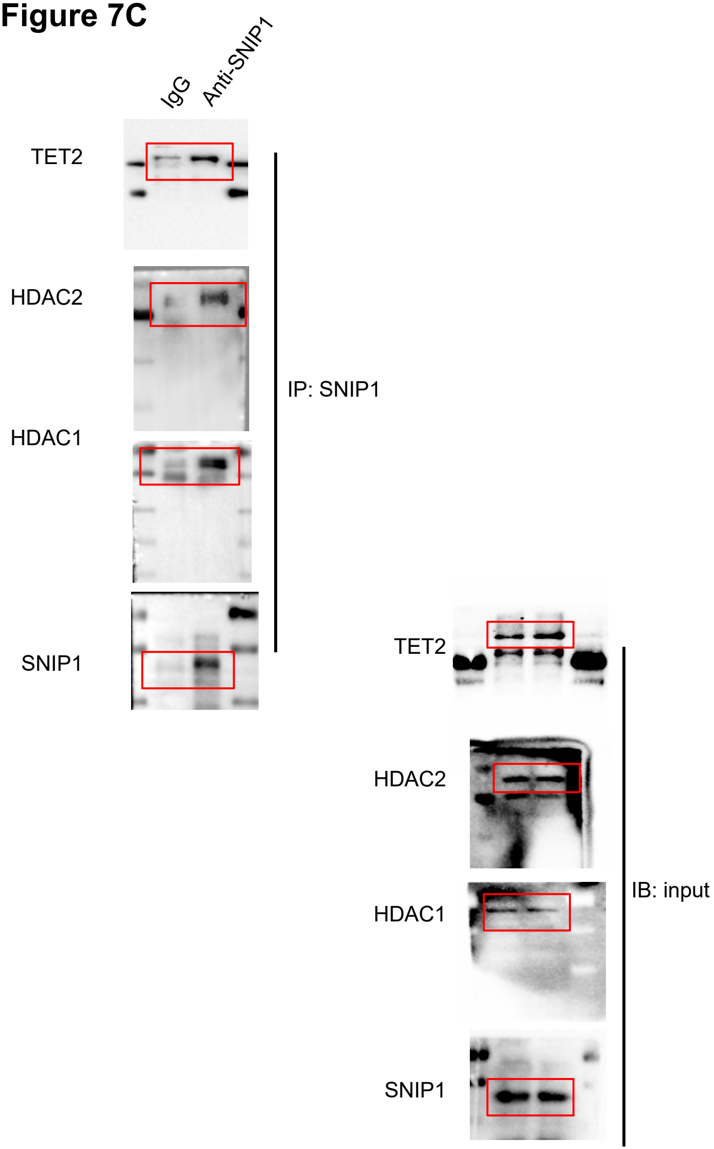

Full unedited gel for Figure 7D

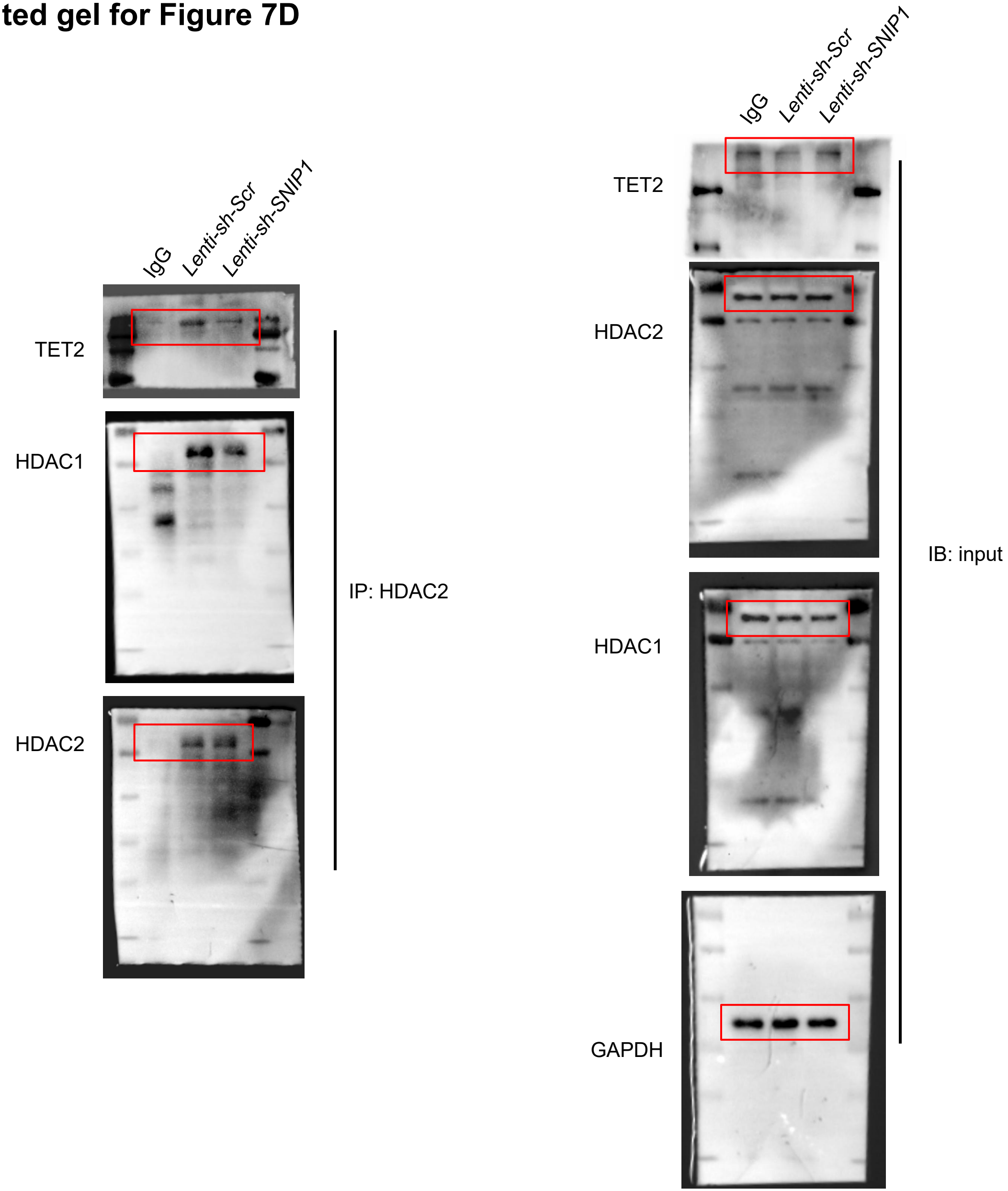

Full unedited gel for Figure 8C

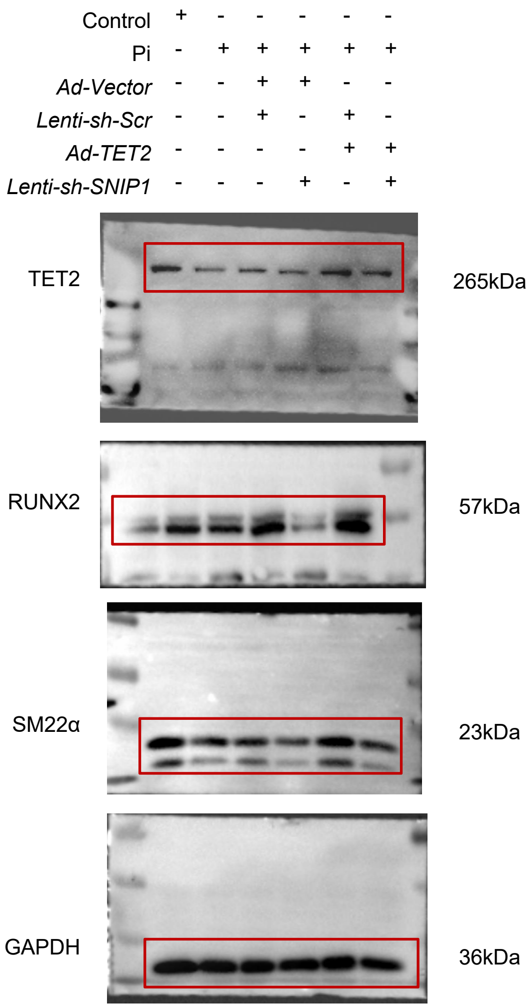

Full unedited gel for Figure 8D

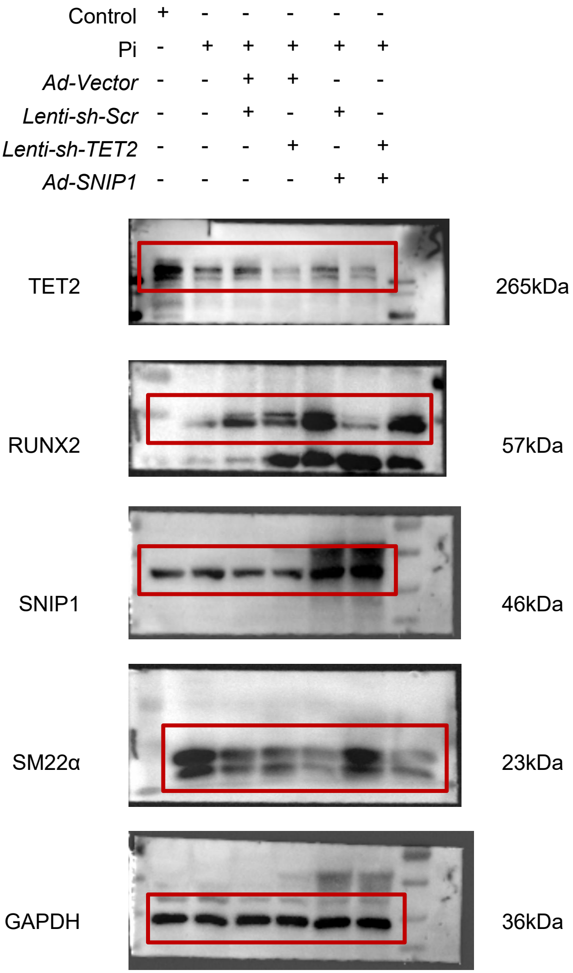

Full unedited gel for Figure 9A and 9D

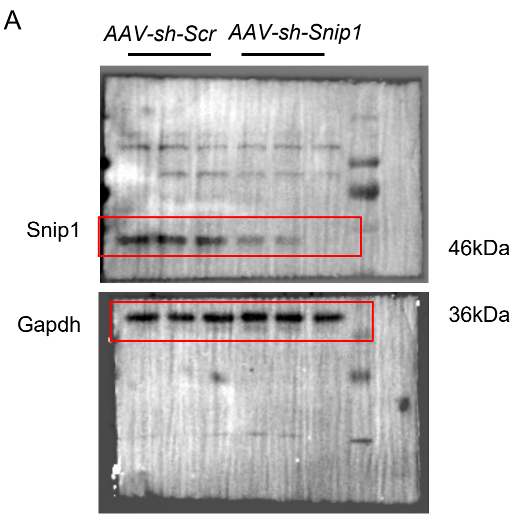

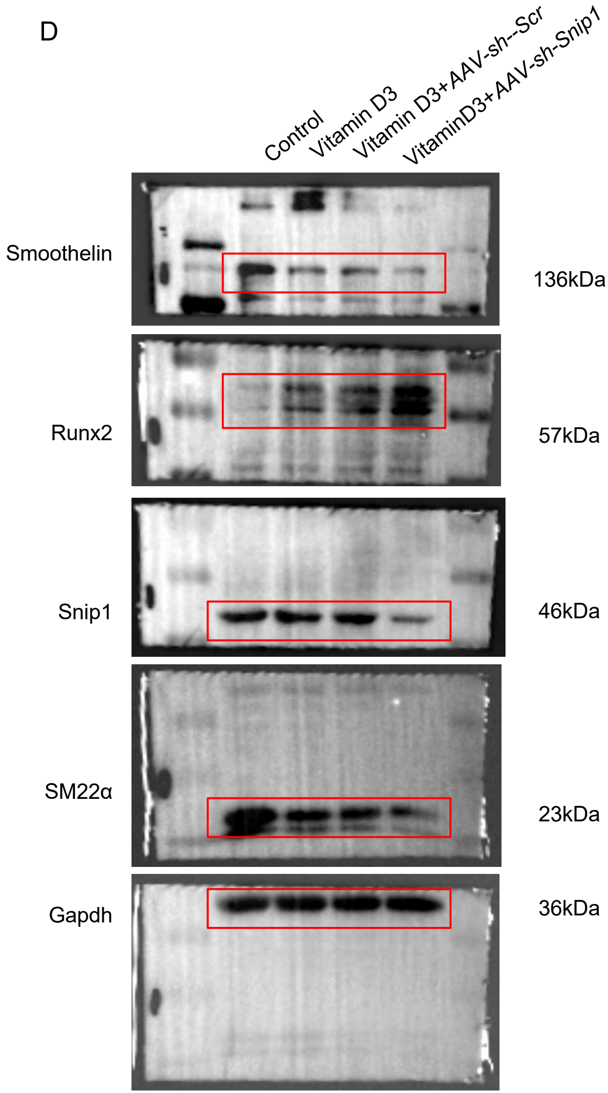

Full unedited gel for Supplemental Figure 3A-E

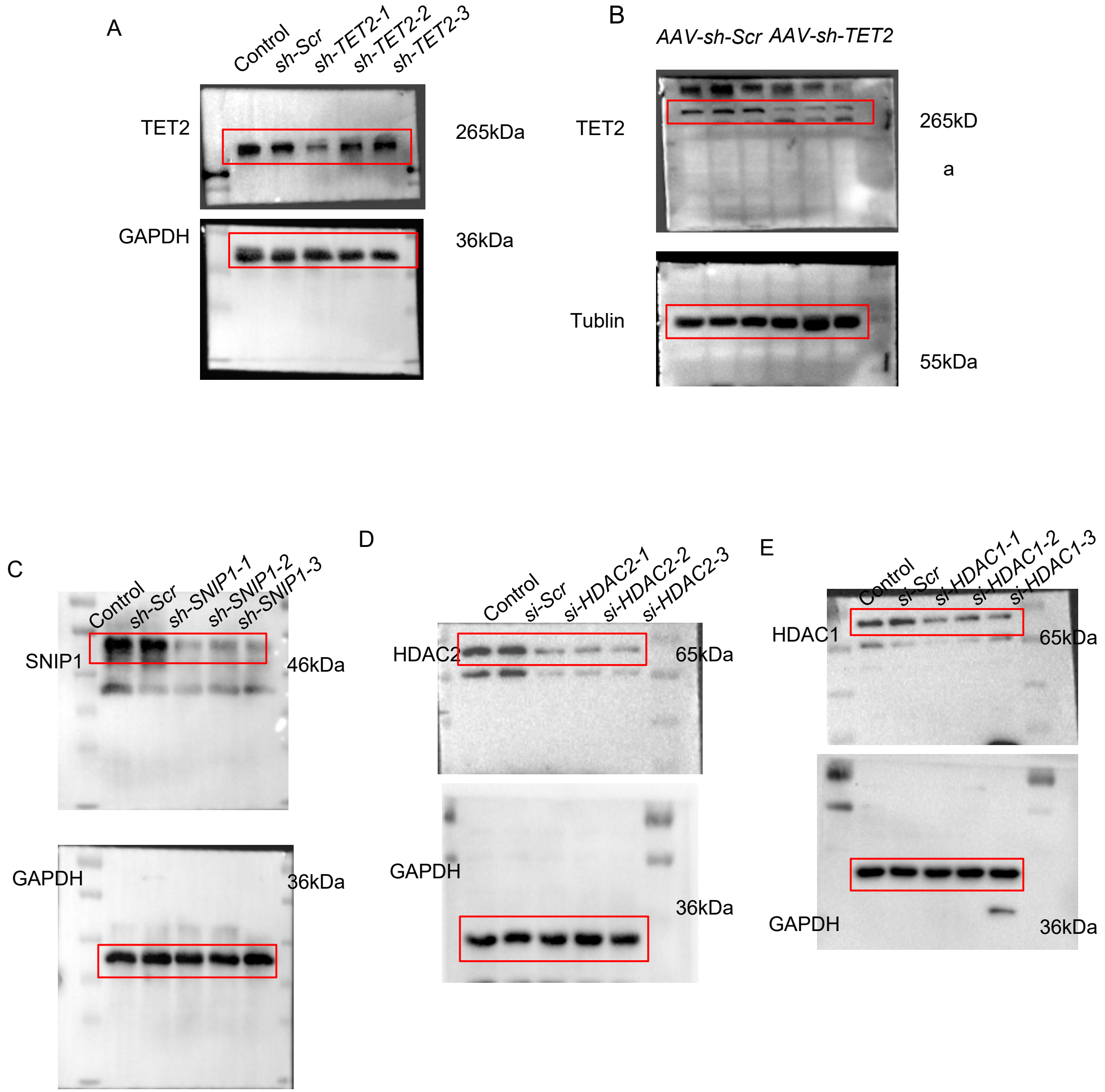

Full unedited gel for Supplemental Figure 4B

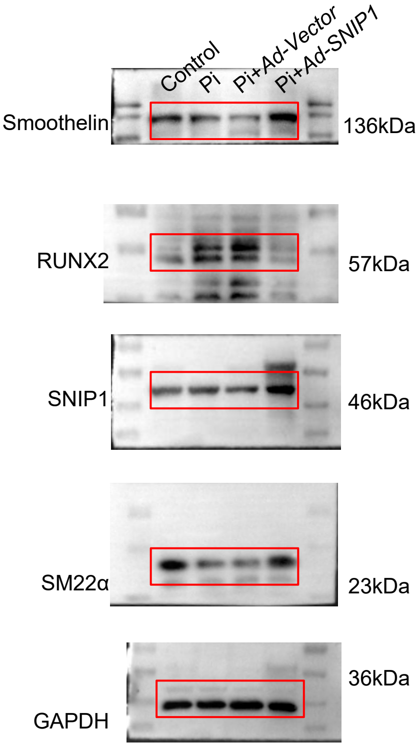

Supplement: Unedited blot and gel images [file jci-135-186673-s127.pdf]
